# Antimicrobial activity of essential oils against multidrug-resistant clinical isolates of the *Burkholderia cepacia* complex

**DOI:** 10.1371/journal.pone.0201835

**Published:** 2018-08-02

**Authors:** Lakshmi Vasireddy, Lewis E. H. Bingle, Mark S. Davies

**Affiliations:** Faculty of Health Sciences and Wellbeing, University of Sunderland, Sunderland, United Kingdom; Laurentian, CANADA

## Abstract

Members of the *Burkholderia cepacia* complex (Bcc) are an important cause of opportunistic or nosocomial infections that may be hard to treat due to a high incidence of multidrug resistance. We characterised a collection of 51 clinical isolates from this complex, assigning them to 18 sequence types using multi-locus sequence type analysis. Resistance to eight commonly used antibiotics was assessed using by using agar-dilution assays to calculate MICs and widespread and heterogeneous multidrug resistance was confirmed, with eight strains proving resistant to all antibiotics tested. Disc diffusion screening of antimicrobial activity of a range of plant essential oils against these Bcc isolates identified six oils with significant activity (lavender, lemongrass, marjoram, peppermint, tea tree and rosewood) and broth microdilution assays indicated that of these lemongrass and rosewood oils had the highest activity, with MIC_50_ values of 0.5% and MIC_90_ values of 1%. Comparison of MIC and MBC values showed that four of these six oils, including lemongrass and rosewood, were bacteriocidal rather than bacteriostatic in their effects. Qualitative analysis of the four bacteriocidal essential oils via GC/MS indicated the presence of 55 different component compounds, mostly monoterpenes. We assessed selected essential oil components as anti-Bcc agents and demonstrated that terpinen-4-ol and geraniol were effective with MICs of 0.125–0.5% (v/v) and 0.125–1% (v/v), respectively. Time-kill studies indicate that these two alcohols are effective against non-growing cells in an efflux-dependent manner. Analysis of bacterial leakage of potassium ions and 260 nm UV-absorbing material on treatment with terpinen-4-ol and geraniol suggested that the observed anti-Bcc activity was a consequence of membrane disruption. This finding was supported by a gas chromatography analysis of bacterial fatty acid methyl esters, which indicated changes in membrane fatty acid composition caused by terpinen-4-ol and geraniol. These essential oils or oil components may ultimately prove useful as therapeutic drugs, for example to treat Bcc infections in CF patients.

## Introduction

The *Burkholderia cepacia* complex (Bcc) is a group of genetically distinct but phenotypically very similar bacteria that were originally isolated as plant pathogens [1]. During the late 1980s Bcc bacteria emerged as an important cause of opportunistic or nosocomial infections [2,3,4]. Bcc organisms are particularly virulent pathogens in cystic fibrosis (CF) patients and are associated with a poor prognosis, a rapid decline in lung function and reduced median survival [5,6]. A significant minority of CF patients become infected with Bcc bacteria at some point, for example a recent study at an Irish adult cystic fibrosis centre suggested a prevalence of around 6% and mortality caused by the most virulent species (*B*. *cenocepacia*) of 55% [7]. Cross-infection with the Bcc between CF patients is important but it is believed that most infections have an environmental origin [8]; all 20 species of the complex can be isolated, in varying frequencies, from CF or non-CF patient sputum and at least 17 of these species can also be isolated from the environment [4,9,10]. The pathogenic potential of the complex varies between species with *B*. *multivorans* and *B*. *cenocepacia* known to be particularly virulent [11].

The heterogeneous intrinsic resistance of Bcc bacteria to a wide range of antibiotics, including the antipseudomonal colistin, has been well documented (e.g. [12,13,14]). Infections are normally treated with a combination of two or three antibiotics, commonly including tobramycin and meropenem, which may be delivered IV, orally or via a nebuliser [15,16]. However, such treatments do not have a good foundation in terms of clinical data [17]. Due to widespread multidrug resistance in Bcc strains, new treatment options are needed [18]. Novel antimicrobial strategies against the Bcc recently proposed have included the use of antisense technologies to modulate essential gene expression and the use of plant-derived essential oils [19,20]. Essential oils (EOs) are complex hydrophobic liquids containing multiple volatile low molecular weight compounds (often terpenes or terpenoids) and obtained by distillation, mechanical expression or solvent extraction from plant matter. Essential oils and essential oil components (EOCs) have been previously shown to have potentially useful antimicrobial activities against gastrointestinal and other pathogens [21,22,23,24]. Recently, 3 essential oils, from *Eugenia caryophyllata* (clove, synonym: *Syzygium aromaticum*), *Origanum vulgare* (oregano) and *Thymus vulgaris* (thyme), were found to be strongly active against Bcc strains, including environmental and clinical strains and antibiotic resistant isolates [20].

This study builds on current knowledge through a novel sequence of four components: characterisation of a collection of clinical isolates of the Bcc in terms of multilocus sequence typing (MLST) and antibiotic susceptibility; assessment of antimicrobial activity of a range of essential oils against these Bcc isolates; assessment of anti-Bcc antimicrobial activity of essential oil components; and characterisation of the mechanism of action of essential oil components against the Bcc.

## Materials and methods

### Bacterial strains

Fifty-one clinical isolates were collected between 2003 and 2007. These were obtained from 41 CF patients (from various parts of the UK) when they visited the Freeman Hospital, Newcastle, UK for lung transplantation. Where multiple isolates of the same species (based on PCR-RFLP analysis) were identified from the same patient, only the first confirmed as from the Bcc was included in the study. Multiple isolates from the same patient belonging to different species were included in the study. As controls, six laboratory strains, from the panel of Bcc strains referred to by Mahenthiralingam *et al*. were used (Table 1)[25]. These control strains were selected to represent different species of the Bcc and were sequenced as reference strains, obtaining 100% identity with the sequences listed in the MLST database. Strain LMG16656 / J2315 (Table 1) is representative of a lineage of *B*. *cenocepacia* rarely isolated from the environment that spreads between CF patients and has a completely sequenced genome [26,27]. The strain is metabolically versatile and possesses numerous virulence and drug resistance functions.

**Table 1:** Control strains.

Accession number	Species	Source / location
LMG 16232	*B*. *vietnamiensis*	CF, Sweden
LMG 18863*	*B*. *cenocepacia*	CF, Canada
LMG 18825*	*B*. *multivorans*	CF, UK
LMG 16656 / J2315*	*B*. *cenocepacia*	CF, UK
LMG 17997	*B*. *cepacia*	Urinary tract infection, Sweden
LMG 18870	*B*. *stabilis*	CF, Canada

CF, Cystic fibrosis; UK, United Kingdom;

* Epidemic strains (strains that can transfer between patients)

### Culture, storage and recovery of isolates

After overnight culture in tryptone soy broth (TSB), cells were pelleted, re-suspended in 2xTSB with 25% (v/v) glycerol and stored at -80°C until required. After thawing Bcc isolates were inoculated on to tryptone soy agar (TSA) and incubated overnight at 37°C. Strain purity was confirmed by growing the cultures on *B*. *cepacia* agar base (BCAB; Oxoid) with *B*. *cepacia* selective supplement (polymixin B, gentamycin, ticarcillin; Oxoid). Working cultures were maintained on TSA slants and subcultured weekly.

### Characterization of the Bcc by MLST

The protocol developed by Sullivan *et al*. was adopted for DNA extraction and automated system performance and sequencing conditions [28]. PCR amplification conditions and primers used were selected based on the information available on the MLST co-ordinating website (http://pubmlst.org/bcc).

Template DNA for PCR was extracted as follows. Between 5 and 10 fresh colonies of an overnight culture were transferred into 0.5 ml distilled water and heated to 100°C for 15 min. The suspension was centrifuged at 15000 × *g* for 2 min and supernatant transferred into a fresh tube. PCR amplification was carried out using a liquid handling robot (TheOnyx, Automated Lab Solutions GmbH). All PCR reagents were maintained at 4°C in the reagent rack. Each PCR was performed in a final volume of 50 μl using a Taq polymerase PCR master mix (ReadyMix, ABgene) with oligonucleotide primers at 0.4 μM and 6 μl of extracted template DNA. Thermal cycling conditions were: initial denaturation at 96°C for 1 min; 30 cycles of denaturation at 96°C for 1 min, primer annealing at 58°C for 1 min, extension at 72°C for 2 min; final extension step of 72°C for 5 min. PCR products were purified using a MultiScreen 384-PCR Filter Plate (Merck Millipore) and eluted twice with a total volume of 70 μl distilled water. Sequencing was carried out on both DNA strands: 6 μl of purified PCR product was mixed with 3 μl of DYEnamic ET terminator sequence premix (Amersham Biosciences) and 10 pmol of each MLST gene primer. Thermal cycling conditions for sequencing reactions were 95°C for 2 min, 30 cycles of 95°C for 20 s, 50°C for 15 s and 60°C for 1 min. Salts and unincorporated dye terminators were removed using a MultiScreen SEQ384 Filter Plate (Merck Millipore). DNA sequencing was performed using an automated MegaBACE 1000 96-capillary sequencer (Amersham Biosciences). DNA sequence data were analyzed and converted into FASTA format using the MegaBACE Sequence Analysis Software with integrated cimaron v1.53 phredify base caller. Sequences were queried against the PubMLST Bcc database (http://pubmlst.org/bcc) to obtain a sequence type (ST).

### Antibiotic susceptibility testing by agar dilution method

Agar dilution assays were performed for 8 commonly-used antibiotics: aztreonam, ceftazidime, ciprofloxacin, colistin, meropenem, tetracycline, tobramycin, and trimethoprim. All antibiotics were purchased from Sigma-Aldrich (UK) except meropenem, purchased from Sequoia Research Products, UK. Antibiotics were serially diluted in 2-fold steps and then 2 ml of each dilution of antibiotic was added to 18 ml of Isosensitest agar (ISA) and mixed thoroughly before pouring. Two control plates containing no antibiotics were prepared; one was used to check the sterility of the medium and the other was used as a growth control. Plates were used within 24 h of preparation.

A standardised inoculum was prepared for each strain tested. After overnight incubation in Isosensitest broth ISB at 37°C, bacteria were collected by centrifugation at 3000 × *g* for 5 min and suspended in sterile distilled water. Turbidity was adjusted to 0.5 McFarland standard by adding sterile distilled water, giving a cell density of ~ 1×10^8^ cfu ml^-1^. This suspension was further diluted by a factor of 10 and then 1 μl of diluted broth culture, providing a final inoculum of approximately 10^4^ cfu, was spot inoculated onto agar plates within 15 min of preparation of the inoculum. Inoculated plates were incubated for 16–18 h at 37°C before determination of MICs. MIC was defined as the lowest concentration at which there was no visible growth.

As Bcc breakpoints were not available for all antibiotics tested against the Bcc, where necessary breakpoints were those advocated by the British Society for Antimicrobial Chemotherapy (BSAC) for the respective method for *Pseudomonas* spp. [29] and for this reason *P*. *aeruginosa* NCTC10662 was included as a reference strain (Bcc members were previously classified in the genus *Pseudomonas*). Also *B*. *cepacia* LMG 16656/J2315 from panel strains of the Bcc [25] was included in the study, allowing better comparison of our results with other studies.

### Essential oils

The EOs used in testing the susceptibility of members of the Bcc were purchased from two different retail suppliers as described in Table 2. As EOs are not certified to a standard composition, batch numbers are given.

**Table 2:** List of essential oils used in this study.

Number	Essential Oil	Plant Species	Supplier	Batch Number
1	Lavender	*Lavandula angustifolia*	Boots UK Limited	67L
2	Geranium	*Pelargonium graveolens*	Boots UK Limited	10L
3	Roman camomile	*Chamaemelum nobile*	Boots UK Limited	15N
4	Tea Tree	*Melaleuca alternifolia*	Boots UK Limited	18L
5	Lemongrass	*Cymbopogon citratus*	Boots UK Limited	44K
6	Peppermint	*Mentha x piperita*	Boots UK Limited	17N
7	Marjoram	*Origanum majorana*	Holland and Barrett Retail Limited	19975
8	Fennel	*Foeniculum vulgare*	Holland and Barrett Retail Limited	18189
9	Pine	*Pinus sylvestris*	Holland and Barrett Retail Limited	20043
10	Cedarwood	*Cedrus atlantica*	Holland and Barrett Retail Limited	19981A
11	Juniper	*Juniperus communis*	Holland and Barrett Retail Limited	19111
12	Frankincense	*Boswellia carterii*	Holland and Barrett Retail Limited	19474
13	Black pepper	*Piper nigrum*	Holland and Barrett Retail Limited	19057
14	Camomile	*Matricaria recutita*	Holland and Barrett Retail Limited	19029
15	Ylang-Ylang	*Cananga odorata*	Holland and Barrett Retail Limited	19252
16	Rosemary	*Rosmarinus officinalis*	Boots UK Limited	17K
17	Bergamot	*Citrus aurantium var*. *bergamia*	Boots UK Limited	21N
18	Sweet orange	*Citrus x sinensis*	Holland and Barrett Retail Limited	19567
19	Rosewood	*Aniba rosaeodora*	Holland and Barrett Retail Limited	19780

### Qualitative analysis of essential oils using GC/MS

The chemical composition of oils was determined by GC/MS using a Trace 2000 GC (Thermo Finnigan) interfaced with a Voyager single quad MS apparatus (Thermo Quest). Parameters are listed in Table 3. Mass spectra and retention times of essential oil components were identified by comparing them with those of authentic standards from the mass spectra of National Institute of Standards and Technology library. Chromatographic profiles of oils were compared against ISO standards as follows: lemongrass, ISO 4718:2004; tea tree, ISO 4730:2004; marjoram, ISO 4728:2003; rosewood, ISO 3761:2005.

**Table 3:** Parameters used in GC/MS analysis of essential oils.

Column	Zebron ZB5-MS
Stationary phase	5%phenyl-95%-Dimethyl polysiloxane copolymer
Dimensions	30 m long 0.25 mm × 0.25 μm
Sample quantity injected	0.5 μl
Carrier gas	Helium at a constant flow of 1 ml min^-1^
Oven profile	50°C to 275°C at 5°C min^-1^. Final temperature held for 5 min
Inlet temperature	250°C
Split ratio	100:1
Ionization mode	Electron impact positive mode
Ion source temperature	250°C
Multiplier	149 V
Mass range analysed	1–300

### Essential oil components tested

Terpenoid standards used in the study and their purity percentages as obtained from Sigma-Aldrich (UK) were: alloaromadendrene (-) (98%), camphene (95%), camphor (R) (95%), caryophyllene (-) (trans) (98.5%), 1,8-cineole, citral (95%), p-cymene (99%), geraniol (98%), geranyl acetate (97%), ledene (+) (95%), limonene (R) (+) (97%), linalool (±) (95%), linalyl acetate (97%), 6-methyl-5-hepten-2-one (96%), myrcene (90%), α-pinene (-) (99%), β-pinene (-) (99%), sabienene hydrate (97%), α-terpinene (85%), γ-terpinene (97%), terpinolene (95%), terpinen-4-ol (95%), α-terpineol (96%), terpinyl acetate (±) (90%) and verbenol (95%).

### Disc diffusion assays

Disc diffusion assays were performed in triplicate for the EOs listed in Table 2 on 57 strains of the Bcc (51 clinical isolates and 6 reference strains) using two media, TSA and ISA, according to BSAC criteria [30]. A standardised inoculum was prepared for each tested Bcc strain as described above for antibiotic susceptibility testing, but the 0.5 McFarland suspension was diluted 1:100 instead of 1:10. Within 15 min of the preparation of the inoculum, the plates were inoculated: 100 μl of diluted bacterial suspension was placed on the agar plate and, using sterile cotton swabs, spread evenly over the entire surface of the agar. Plates were allowed to dry for 10 min then, in triplicate, 5 μl of each neat EO was pipetted onto a sterile 6 mm diameter filter paper (Whatman No 4) disc and applied to the agar surface. Assay plates were incubated at 37°C for 24 h. For each disc, the diameter of a zone of inhibition was measured and a zone of diameter > 7 mm was considered as positive.

### MIC and MBC assay using broth microdilution method

The MICs of the six most active EOs (Table 4) were determined against the clinical and reference isolates using the broth microdilution method, essentially as described by BSAC [30]. Broth cultures of each strain were prepared at a concentration of approximately 1×10^8^ cfu/ml as described previously. Tween80 was added to ISB at a final concentration of 0.001% (v/v) to emulsify the EOs [21]. EOs were added to ISB containing Tween80 to give a final EO concentration of between 8% and 0.125%. The contents of each tube were vortex mixed at high speed for 30 s to ensure that the EO was evenly dispersed throughout the broth. 100μl of bacterial culture and 100 μl of EO dilution were then added to the wells of a 96-well microtitre plate. After the bacteria were incubated for 24 h, the MIC was determined by observation of the lowest concentration where there was no visible growth. Subcultures of 10 μl were taken from the clear wells of the microtitre tray (and from control wells) and spot-inoculated onto ISA allowing determination of minimum bacteriocidal concentrations (MBC; the lowest concentration at which no growth on antibiotic-free medium was observed).

**Table 4:** Antimicrobial activities for selected EOs.

Essential oil	Mean ^a^	Standard Deviation	Mean MIC ^a^^,^^b^	Mean MBC ^a^^,^^b^	MIC_50_ ^b^	MBC_50_ ^b^	MIC_90_ ^b^	MBC_90_ ^b^
Teatree	12.4	1.8	1.9	3.8	2	4	2	8
Lemongrass	14.0	3.0	0.7	2.2	0.5	2	1	4
Lavender	10.4	1.5	1.5	7.1	2	>8	2	>8
Marjoram	12.0	2.0	1.8	2.7	2	2	4	4
Rosewood	15.1	3.0	0.5	1.3	0.5	2	1	4
Peppermint	12.5	2.7	1.4	5.2	2	8	2	>8

a) Mean of 51 clinical and 6 control strains.

b) Minimum inhibitory and bactericidal concentrations (% v/v)

MIC_50_ and MIC_90_ [31] were calculated by ranking MICs for all 57 clinical and reference strains in ascending order. The MIC_50_ was taken as the median value. The MIC_90_ was the value of the 51^st^ (0.9 x 57) in the list, and represented the concentration of antibiotic that would inhibit 90% of the isolates tested.

MICs and MBCs for each of the EOCs listed above were determined for all strains using the broth dilution assay as described previously. Stock solutions were prepared in ethanol and vortexed thoroughly for 30 s. The maximum concentrations of camphor and camphene tested were 1% (v/v) while for other compounds this was 8% (v/v). The final concentration of ethanol in the broth was never more than 1% (v/v). Aromadendrene and ledene were tested on the reference strains only (and showed no inhibitory effect).

### Time kill studies in presence and absence of a carbon source

A plate-count-based method was adopted to allow sensitive assessment of viability, since Hershberger et al. [32] showed no difference between time-kill experiments performed using a 24-well plate method and more traditional test tube methods. To 1 ml of ISB in a 24-well tissue culture plate, to which the MBC concentration of terpinen-4-ol or geraniol had already been added, 1 ml of a 1:100 dilution of a 0.5 McFarland standard suspension of either stationary or mid-exponential phase culture (final concentration of 5x10^5^ cfu ml^−1^) was added. The plates were then incubated at 37°C. Samples (50 μl) were removed after 0, 30, 60, 120, 240 min incubations; at each time point an undiluted sample (20 μl) and ten-fold dilutions (10^−1^, 10^−2^, 10^−3^, and 10^−4^) prepared in saline were spread directly onto ISA plates with a micropipette. Plates were incubated for 24 h and the number of surviving organisms (log_10_ cfu ml^−1^) was determined. Each experiment was carried out six times using the same culture with a single replicate plate (three wells and double sampling from each well in a plate) generated at each sampling time.

For time kill studies in the absence of a carbon source the procedure above was followed, except that cells and EOCs were suspended in phosphate buffered saline (PBS) rather than a nutrient medium.

### Time kill studies in the presence of an outer membrane permeabilizer

EDTA was added to the 1 ml of broth containing terpinen-4-ol or geraniol described in the previous section, so that a final concentration of 10 mM EDTA was present. Terpinen-4-ol was tested at a concentration of 0.125% (v/v) and geraniol at 0.25% (v/v); concentrations chosen based on the MICs of the EO components in the presence of EDTA. Cells not treated with EDTA and cells treated with EDTA alone were included as controls. The incubation, collection of samples and plate counts were carried out in the same manner as that of time kill studies in the presence of a carbon source.

### Time kill studies in the presence of an efflux inhibitor

To the 1 ml cell suspension (see above) 1ml of ISB containing double the inhibitory concentration of either terpinen-4-ol or geraniol (0.25% and 0.5%, v/v respectively), carbonyl cyanide m-chlorophenylhydrazone (CCCP), a protonophore that inhibits energy production in cells was added to produce a final concentration of 250 μM. The incubation, collection of samples and plate counts were carried out in the same manner as that of the time kill studies in the presence of a carbon source (see above).

### Assessment of membrane damage by analysis of potassium leakage

Loss of potassium ions was studied according to the method of Codling *et al*. [33]. Bacteria cultured on TSA slants were streaked onto freshly prepared TSA plates and incubated overnight at 30°C, then harvested by centrifugation (3000 × *g* for 15 min) and washed three times with ultra pure water (Elgastat UHP filtered deionized water). Bacteria were adjusted to 2 mg dry weight of cells per ml in ultra pure water. Terpinen-4-ol and geraniol solutions were prepared at double the required concentration (0.5% and 1% (v/v), respectively), so that the addition of an equal volume of cell suspension produced a final concentration of 1 mg dry weight of cells per ml. The bacteria were exposed to terpinen-4-ol and geraniol at 37°C and 10 ml samples were removed at 0, 30, 60, 120, 240 min. Cells were removed by filtration using 0.2 μm pore filters and the filtrates were analysed for K^+^ content using atomic absorption spectrophotometry. EOC-free controls with and without Tween80 were prepared under the same conditions to determine normal K^+^ flux over the time course of the experiment. The positive control was a lysed cell suspension obtained by thermal shock (80°C for 30 min). Experiments were conducted in triplicate. The potassium concentration in the supernatant was measured using an atomic absorption spectrophotometer (Philips PU9200X). The instrumental parameters were as follows: potassium hollow cathode lamp, wavelength 766.5 nm; fuel and flow, acetylene at 2 l min^-1^; oxidant and flow, air at 10 l min^-1^; type of flame, slightly oxidizing; monochromator, 1.8/1.6.

### Leakage of UV (260 nm)-absorbing material

Cells from the working culture (100ml) of *B*. *cenocepacia* were collected by centrifugation (3000 × *g* for 15 min), washed three times with PBS, and resuspended in PBS. Terpinen-4-ol and geraniol were added to the cell suspension at final concentrations equivalent to their MBCs and then the suspension was incubated at 37°C with agitation. Samples (10 ml) were then taken at 0, 30, 60, 120 and 240 min and filtered through a 0.2 μm pore filter. The concentration of the constituents released was determined by UV absorption measurements of each filtrate at 260 nm. Results were expressed as percentages of 260 nm-absorbing material in each interval with respect to the total release by heat treatment (as described above). The experiment was carried out with three replicates for each sample. Cells treated only with Tween80 were also included to study the effect of this surfactant on the leakage of cellular constituents.

### Membrane fatty acid analysis

Bacteria from the working culture were incubated for 24 h at 28°C on TSA with and without terpinen-4-ol or geraniol. Fatty acids were determined as methyl esters from whole-cell hydrolysates, according to the procedure of the SHERLOCK microbial identification system [34]. 40 mg of cell dry mass was subjected to derivatization in screw-cap Teflon test tubes. For the preparation of esters, cells were saponified at 100°C for 30 min with 1 ml of reagent I (45 g of sodium hydroxide, 150 ml of methanol, 150 ml of distilled water), methylated at 80°C for 10 min with reagent II (325 ml of 6 M hydrochloric acid, 275 ml of methanol), extracted with 1.25 ml of reagent III (200 ml of *n*-hexane, 200 ml of methyl *tert*-butyl ether), and base washed with 3 ml of reagent IV (0.27 M sodium hydroxide, 0.68 M sodium chloride). The fatty acid methyl esters (FAME) were analysed by GC using an Agilent 5890 autosampler gas chromatograph, equipped with a DB-WAX column; parameters are shown in Table 5. The FAME peaks were identified by comparison to those of a standard FAME solution (Supelco, 37 component FAME standards) and were integrated using the Varian Star Chromatography Workstation version 5 (Varian Inc.).

**Table 5:** Gas chromatography parameters for FAME analysis.

Column	DB-WAX; 30 m×0.225 mm×0.25 µm
Inlet temperature	260°C
Detector temperature	310°C
Injection volume	0.5μl
Carrier gas and Flow rate	Nitrogen 30 cm s^-1^
Oven programme	140°C to 260°C at 4°C min^-1^. Initial and final temperatures held for 5 min

## Results

### Genotyping of clinical isolates

MLST data were obtained for the 51 clinical strains and used to assign them to 11 different species groups and 18 sequence types (STs, Table 6). There were no novel Bcc ST types when compared with existing MLST data (PubMLST, http://pubmlst.org/bcc). The STs identified in the collection were either previously reported from CF patients (STs: 3, 15, 25, 31, 40, 72, 97, 116, 125, 206, 240, 267, 319) or from the environment (3, 88, 125, 154, 165, 290, 291). Three STs (15, 116, 206) identified in the collection were also previously reported from UK CF patients. In the PubMLST data set, ST 88 was reported only from the UK environment, but the present study reported isolation from a CF patient. ST40 was reported from a non-CF patient from the UK and from CF patients in other countries. Average nucleotide identity analysis of the housekeeping genes used in MLST indicated that all strains provisionally identified as “other species” were most similar (greater than 96% ANI) to *B*. *cenocepacia*.

**Table 6:** Species and sequence type (ST) for Bcc clinical isolates.

Species group	Number (%)	ST	Number
*B*. *cenocepacia* IIIA	9 (17.6)	31	9
*B*. *cenocepacia* IIIB	10 (19.6)	40	1
		125	6
		267	3
*B*.*cenocepacia* IIID	1 (2.0)	240	1
*B*. *dolasa*	3 (5.9)	72	3
*B*. *vietnamiensis*	7 (13.7)	319	7
*B*. *multivorans*	3 (5.9)	15	1
		25	2
*B*. *ambifaria*	3 (5.9)	165	1
		290	1
		291	1
*B*. *anthina*	1 (2.0)	88	1
*B*. *cepacia*	1 (2.0)	3	1
*B*. *contaminans*	1 (2.0)	97	1
Other Bcc	12 (23.5)	116	5
		154	3
		206	4

### Antibiotic resistance of clinical isolates

The library of Bcc clinical isolates was tested for resistance to a range of commonly used antibiotics: aztreonam, ceftazidime, ciprofloxacin, colistin, meropenem, tetracycline, trimethoprim and tobramycin. MICs were obtained for each antibiotic and each strain (S1 Table). These data indicated that resistance is strain-specific, irrespective of genomovar / species status (Table 7). MIC values were converted into susceptibility categories (susceptible; intermediate; resistant) according to breakpoints as described for *Pseudomonas* spp. (Table 8; [29]). While BSAC’s most recent advice is that it is not possible to establish clinically-relevant MIC breakpoints for the Bcc (http://bsac.org.uk/wp-content/uploads/2014/06/Burkholderia-Susceptibility-Testing-final.pdf), the Clinical and Laboratory Standards Institute has recently supplied such guidelines [35] for two of these antibiotics (ceftazidime and meropenem) and these breakpoints were used in Table 8. Bcc isolates are commonly multiply antibiotic resistant and intrinsically resistant to colistin. All 51 of our isolates were resistant to tobramycin and colistin, while a majority were resistant to all other antibiotics tested except meropenem (22% of isolates resistant). Ceftazidime and aztreonam also had some activity against the Bcc strains: 43% and 33% of strains were susceptible, respectively. Ciprofloxacin and tetracycline were only active, to an intermediate or susceptible levels respectively, against single (and different) ST206 strains. Although trimethoprim was active against only two strains, an intermediate level of activity was shown on nineteen strains. Meropenem was active against the largest number of strains compared to other antibiotics tested. The mean level of resistance (i.e. the proportion of the 8 antibiotics tested that strains were resistant to) was 75% ± 17 SD. Eight strains belonging to *B*. *cenocepacia*, *B*. *multivorans* and *B*. *dolasa* exhibited 100% resistance.

**Table 7:** Antibiotic resistance of 51 Bcc clinical isolates. Number of isolates resistant to a given antibiotic is shown for each genomovar (percentage of isolates in brackets).

Genomovar	N	Tobra	Cipro	Trimet	Mero	Colis	Tet	Ceftaz	Aztreo
*B*. *cenocepacia* IIIA	9	9 (100)	9 (100)	4 (44)	1 (11)	9 (100)	9(100)	4 (44)	4 (44)
*B*. *cenocepacia* IIIB	10	10 (100)	10 (100)	6 (60)	3 (30)	10 (100)	10 (100)	7 (70)	8 (80)
*B*.*cenocepacia* IIID	1	1 (100)	1 (100)	1 (100)	1 (100)	1 (100)	1 (100)	1 (100)	1 (100)
*B*. *dolasa*	3	3 (100)	3 (100)	3 (100)	2 (67)	3 (100)	3 (100)	3 (100)	3 (100)
*B*. *vietnamiensis*	7	7 (100)	7 (100)	3 (43)	1 (14)	7 (100)	7 (100)	2 (29)	3 (43)
*B*. *multivorans*	3	3 (100)	3 (100)	1 (33)	1 (33)	3 (100)	3 (100)	3 (100)	3 (100)
*B*. *ambifaria*	3	3 (100)	3 (100)	3 (100)	0 (0)	3 (100)	3 (100)	1 (33)	3 (100)
*B*. *anthina*	1	1 (100)	1 (100)	1 (100)	0 (0)	1 (100)	1 (100)	0 (0)	0 (0)
*B*. *cepacia*	1	1 (100)	1 (100)	1 (100)	0 (0)	1 (100)	1 (100)	1 (100)	1 (100)
*B*. *contaminans*	1	1 (100)	1 (100)	0 (0)	0 (0)	1 (100)	1 (100)	0 (0)	1 (100)
Other Bcc	12	12 (100)	11 (92)	7 (58)	2 (17)	12 (100)	11 (92)	5 (42)	7 (58)
**Total resistant**		**51 (100)**	**50 (98)**	**30 (59)**	**11 (22)**	**51 (100)**	**50 (98)**	**27 (53)**	**34 (67)**
**Total intermediate**		**0**	**1 (2)**	**19 (37)**	**13 (25)**	**0**	**0**	**2 (4)**	**0**
**Total sensitive**		**0**	**0**	**2 (4)**	**27 (53)**	**0**	**1 (2)**	**22 (43)**	**17 (33)**

Tobra, tobramycin; Cipro, ciprofloxacin; Trimeth, trimethoprim; Mero, meropenem; Colis, colistin; Tet, tetracycline; Ceftaz, ceftazidime; Aztreo, aztreonam.

**Table 8:** MIC breakpoints (mg/l) used to assign susceptibility categories.

Antibiotic	Susceptible	Intermediate	Resistant
Tobramycin	**≤** 4	-	**>** 4
Ciprofloxacin	**≤** 0.5	1	**>** 1
Colistin	**≤** 2	-	**>** 2
Trimethoprim	**≤** 0.5	-	**>** 4
Meropenem	**≤** 4	8	≥ 16
Aztreonam	**≤** 4	-	**>** 8
Ceftazidime	**≤** 8	16	≥ 32
Tetracycline	**≤** 1	-	**>** 2

### Disc diffusion screening of essential oils for anti-Bcc activity using two different media, TSA and ISA

Fifteen EOs had some activity against the tested strains on ISA. To examine the possibility of medium-specific effects all EOs were also tested on TSA and only one positive effect on ISA that was not reproduced on TSA was noted, for geranium EO against a clinical strain. In general more activity was seen on TSA than on ISA, i.e. additional positives against clinical isolates were seen for TSA: 6 for juniper, 2 for geranium, 5 for rosemary. Therefore results presented here (Table 9) are for ISA as these are more likely to be robust / medium independent. Of the fifteen EOs that showed activity, six were most active against Bcc members, showing activity on all strains tested: lavender, lemongrass, marjoram, peppermint, tea tree and rosewood (Table 9). Fennel, geranium, juniper and rosemary were active against some but not all strains. Pine, black pepper, rosemary, sweet orange, camomile, cedarwood, bergamot, ylang ylang and frankincense—were ineffective against all Bcc members tested. The six most active EOs were selected for further study (Table 9). As with antibiotics, activity of these EOs was highly strain-specific and no consistent patterns of genomovar / species level differences were observed.

**Table 9:** Number of clinical strains showing mean zones of inhibition > 7mm on ISA.

Essential oil	Clinical strains (n = 51)	Reference strains (n = 6)
Lavender	51	6
Lemongrass	51	6
Tea tree	51	6
Rosewood	51	6
Marjoram	51	6
Peppermint	51	6
Geranium	37	6
Fennel	35	6
Rosemary	29	6
Juniper	21	1
Frankincense	0	0
Black pepper	0	0
Camomile	0	0
YlangYlang	0	0
Bergamot	0	0
Sweet orange	0	0
Cedarwood	0	0
Pine	0	0

### MIC and MBC by broth microdilution method

Fifty-one strains of clinical origin and six strains from the Bcc reference panel were tested for susceptibility to the six most active EOs chosen from the disc diffusion assay (Table 9). MICs and MBCs are summarised in Table 10 and listed for each strain in S2 Table. The MIC_50_ (minimum concentration required to inhibit half of strains tested) for lemongrass and rosewood oils was 0.5% (5 mg / ml), indicating Bcc members were highly susceptible to these oils. Marjoram, lavender, tea tree and peppermint had a similar but lower efficiency (2%; 20 mg /ml) against 50% of the Bcc strains. The concentration required to inhibit 90% of the strains tested (MIC_90_) ranged from 1% (10 mg /ml; lemongrass and rosewood) to 4% (40 mg / ml; marjoram). For all EOs except lavender and peppermint, mean MBC, MBC_50_ and MBC_90_ values were less than 4x higher than the corresponding MIC values and therefore these oils can be defined as bacteriocidal rather than bacteriostatic [35,36].

**Table 10:** Minimum inhibitory and bactericidal concentrations (% v/v) for two essential oil components, terpinen-4-ol and geraniol, against clinical isolates and control strains of the Bcc.

EO component	Mean MIC ^a^	Mean MBC ^a^	MIC_50_	MBC_50_	MIC_90_	MBC_90_
Geraniol	0.4	0.4	0.5	0.5	1.0	1.0
Terpinen-4-ol	0.4	0.4	0.5	0.5	0.5	0.5

a) Mean of 51 clinical and 6 control strains.

### Analysis of essential oil components

Qualitative analysis of the EOs marjoram, tea tree, rosewood and lemongrass, enabled the detection of 55 different components that had relative abundances greater than 10% and / or were flagged by the instrument (S3, S4, S5 and S6 Tables). The principal components for each oil were identified as follows: marjoram, eucalyptol; tea tree, terpinen-4-ol; rosewood, linalool; lemongrass, α-citral. Similarity index (SI) and reverse similarity index (RSI) mass spectral match factors for all the compounds except dihydrolinalool were above 800, allowing good identification of compounds.

The majority of EO constituents were monoterpenes, including: monoterpene alcohols such as terpinen-4-ol or geraniol; bicyclic monoterpenes such as camphene, borneol, pinenes, sabinene or camphor; acyclic monoterpenoids (or derivatives) such as myrcene, geranyl acetate, citronellol or linalool. Several sesquiterpenes such as cadinene, caryophyllene, caryophyllene oxide or globulol were also present. Some compounds such as pinene, limonene, citrol, dihydrolinalool, α-terpineol, terpiene-4-ol, p-cymene, myrcene and 1,8-cineole were present in more than one oil.

### Susceptibility of the Bcc to essential oil components

A range of EO components, present in the bacteriocidal oils, was tested against the Bcc test panel: alloaromadendrene (-), camphene, camphor (R), caryophyllene(-)(trans), 1,8-cineole, citral, geraniol, geranyl acetate, ledene(+), limonene (+) (R), linalool (±), linalyl acetate, 6-methyl-5-hepten-2-one, myrcene, α-pinene(-), β-pinene(-), p-cymene, sabienene hydrate, α-terpinene, γ-terpinene, terpinolene, terpinen-4-ol, α-terpineol, terpinyl acetate (±), verbeneol. Only two of the essential oil components tested at concentrations of 8% (v/v) or less showed any activity against the investigated strains: terpinen-4-ol, which is the main water soluble component of tea tree oil and also present in marjoram oil ([37] and S3 and S4 Tables) and geraniol, which is found in many EOs although not detected in any of those analysed here (the derivative geranyl acetate was a major component of our lemongrass oil, but was not found to be active against the Bcc). Both terpinen-4-ol and geraniol showed activity against all the strains tested (S7 Table): MICs ranged from 0.125–0.5% (v/v) and 0.125–1% (v/v), respectively; summary statistics are shown in Table 10. For the first time, terpinen-4-ol and geraniol were found to inhibit Bcc strains.

### Characterisation of geraniol and terpinen-4-ol mechanisms of action against Bcc using time-kill studies in the presence and absence of a carbon source

**Fig 1:**
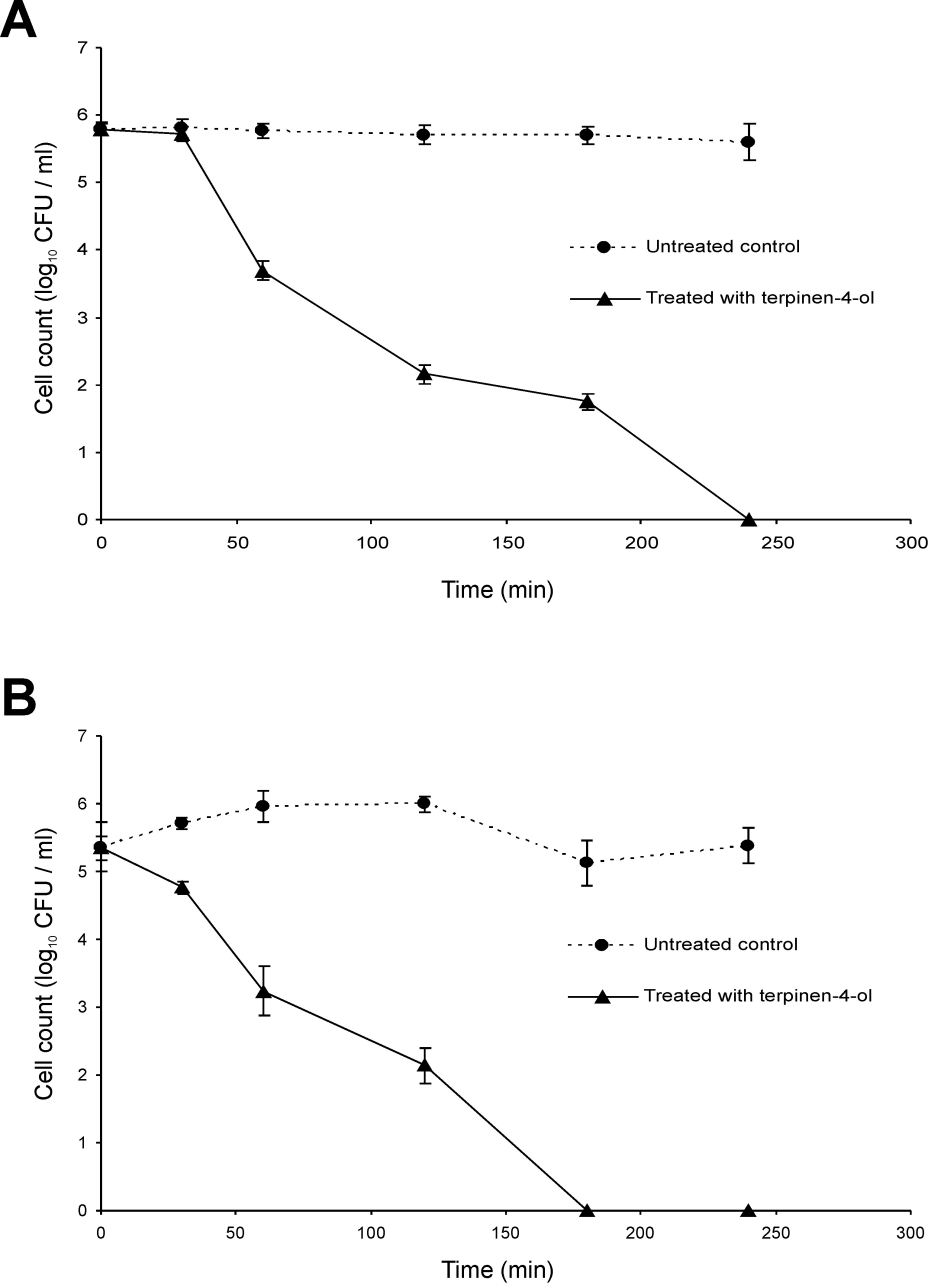
Time-kill analysis of *Burkholderia cenocepacia* strain LMG 16656 when exposed to terpinen-4-ol (0.25% v/v). Cultures were grown to (A) stationary phase or (B) mid-exponential phase. Cell counts are mean values (n = 6); error bars show standard deviation.

**Fig 2:**
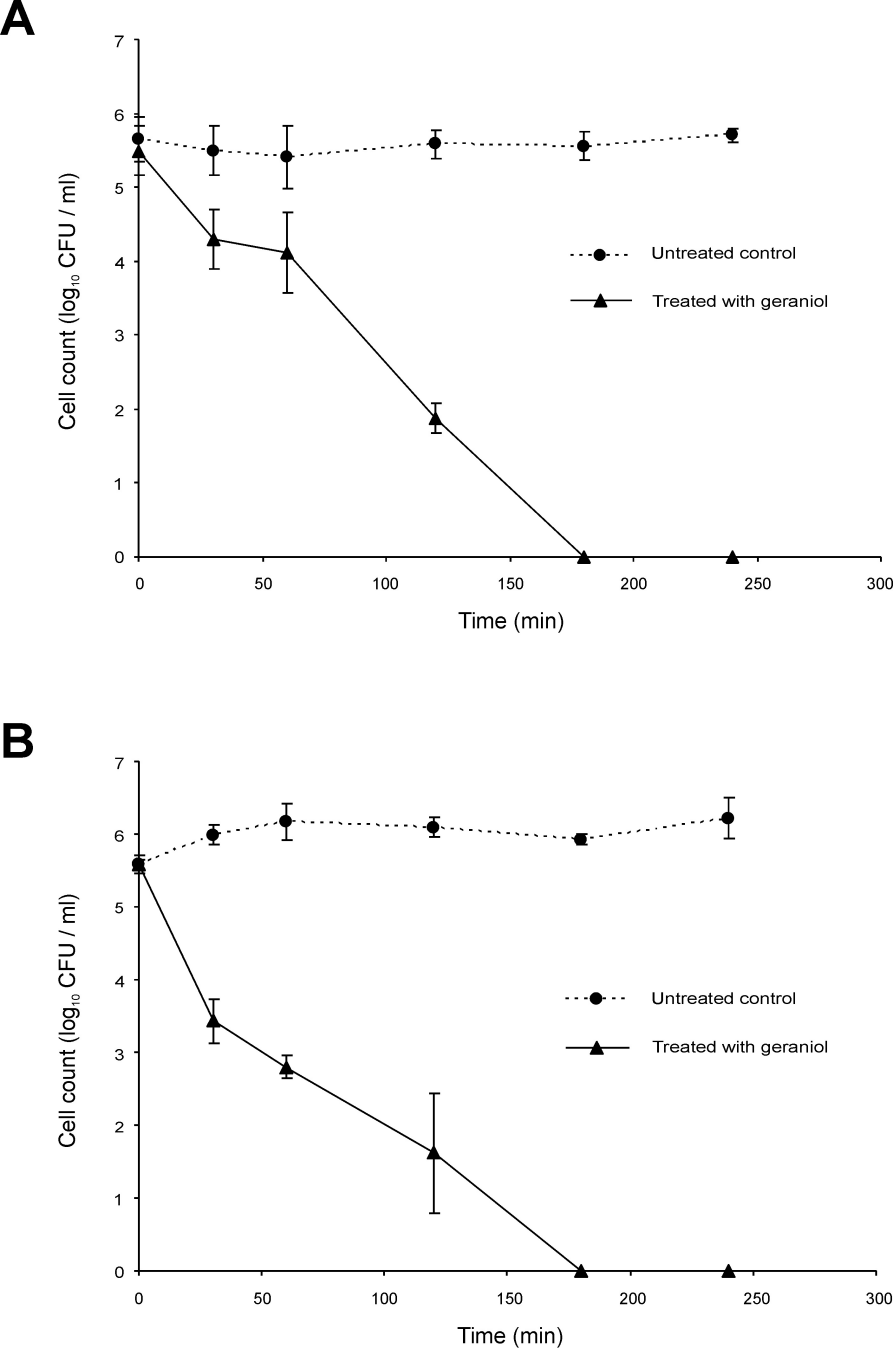
Time-kill analysis of *Burkholderia cenocepacia* strain LMG 16656 when exposed to geraniol (0.5% v/v). Cultures were grown to (A) stationary phase or (B) mid-exponential phase. Cell counts are mean values (n = 6); error bars show standard deviation.

Bactericidal activity for most antibiotics depends on the bacterial growth phase, often requiring ongoing metabolic activity and division of bacterial cells [38]. Whether this is true for the EO components geraniol and terpinen-4-ol, however, is unknown. The kill-rate for cells in two different growth phases was therefore studied: exponential and stationary phase populations of *B*. *cenocepacia* strain LMG 16656 were challenged. Compared with the killing curves observed for stationary phase cultures treated with terpinen-4-ol (0.25%), log phase cultures are killed slightly more swiftly (Fig 1). Approximately 180 min in contact with terpinen-4-ol was required to kill all log phase cells, whereas 240 min was required to completely eradicate *B*. *cenocepacia* in the stationary phase. In the case of geraniol (0.5%) treatment, complete kill was observed after 180 min for both growth phases (Fig 2). After 30 and 60 min, however, stationary phase cultures experienced approximately only 1 log_10_ reduction, whereas log phase cultures at the same time points reduced to 2 log_10_ and 3 log_10_, respectively.

**Fig 3:**
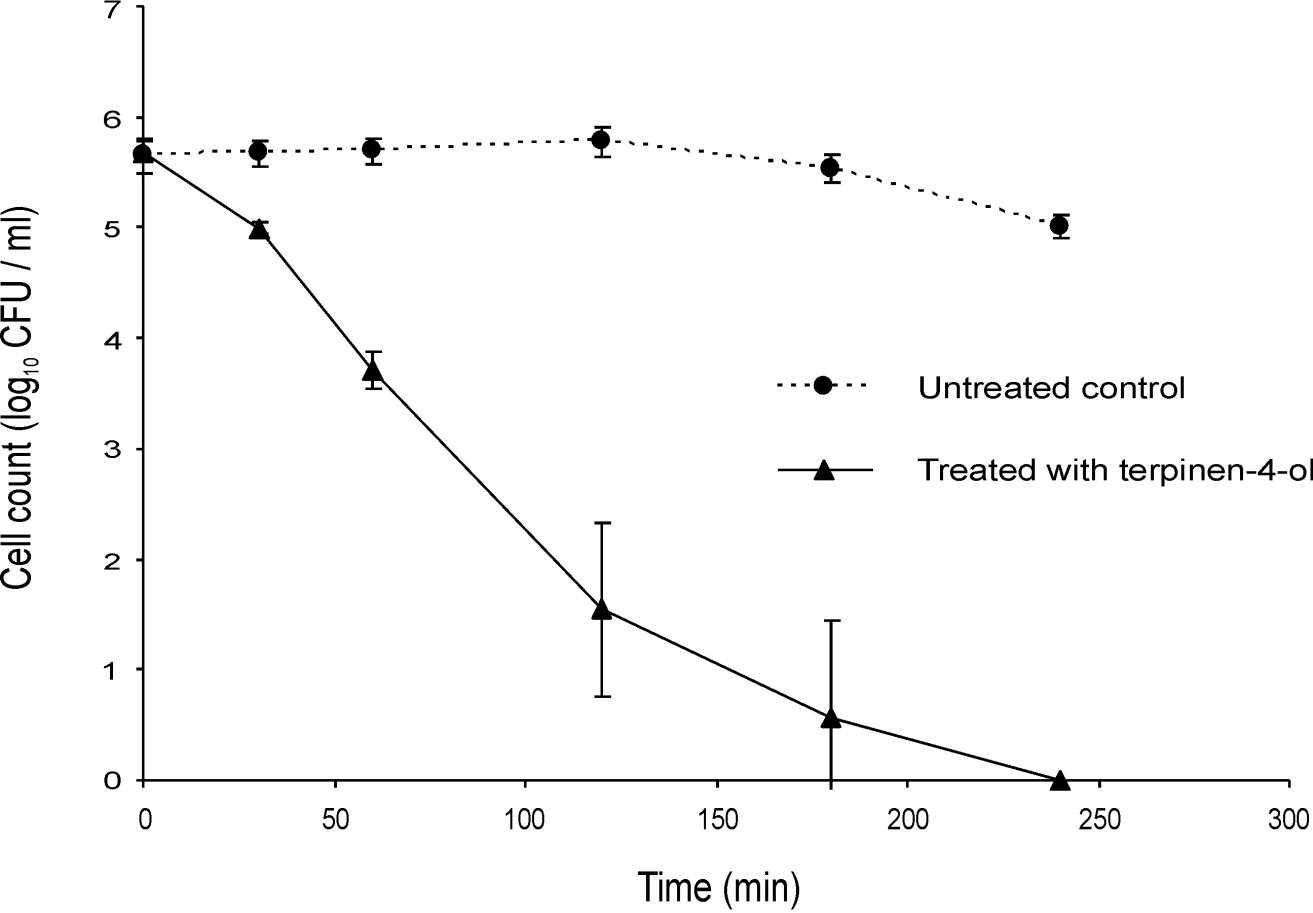
Time-kill analysis of a mid-exponential phase culture of *Burkholderia cenocepacia* strain LMG 16656 when exposed to terpinen-4-ol in PBS (0.25%, v/v). Cell counts are mean values (n = 6); error bars show standard deviation.

**Fig 4:**
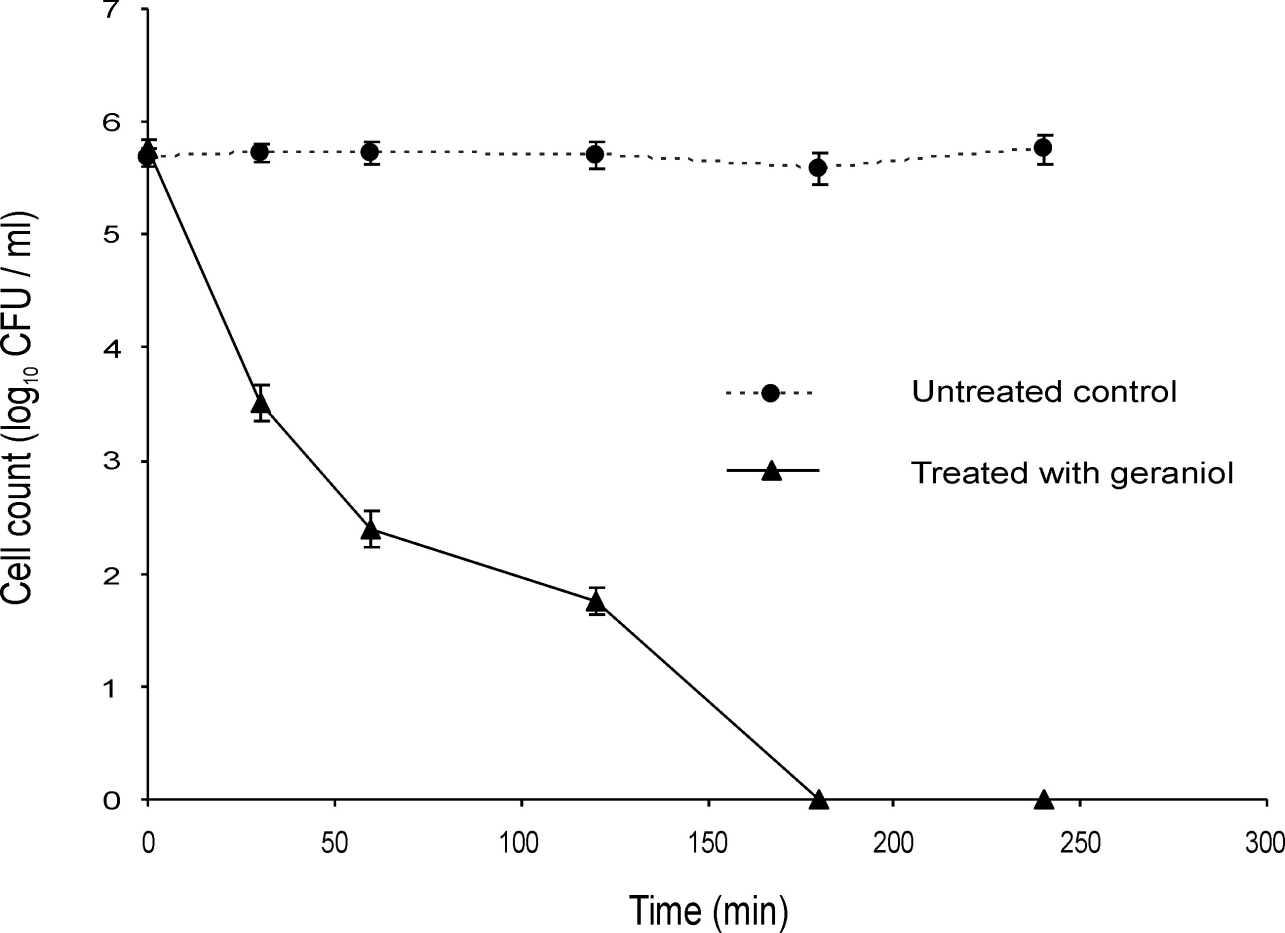
Time-kill analysis of a mid-exponential phase culture of *Burkholderia cenocepacia* strain LMG 16656 when exposed to geraniol (0.5%, v/v) in PBS. Cell counts are mean values (n = 6); error bars show standard deviation.

If these EOCs are inhibiting metabolic processes they may not be active against populations that are not growing, for example when suspended in PBS rather than a nutrient medium such as ISB. There was no noticeable difference in the decline of mid-exponential phase cells of the Bcc upon challenge with terpinen-4-ol (Fig 3) and geraniol (Fig 4) in PBS compared with the same challenge in ISB (Figs 1 and 2).

### Characterisation of geraniol and terpinen-4-ol mechanisms of action against Bcc using time-kill studies in the presence of an outer membrane permeabilizer

**Fig 5:**
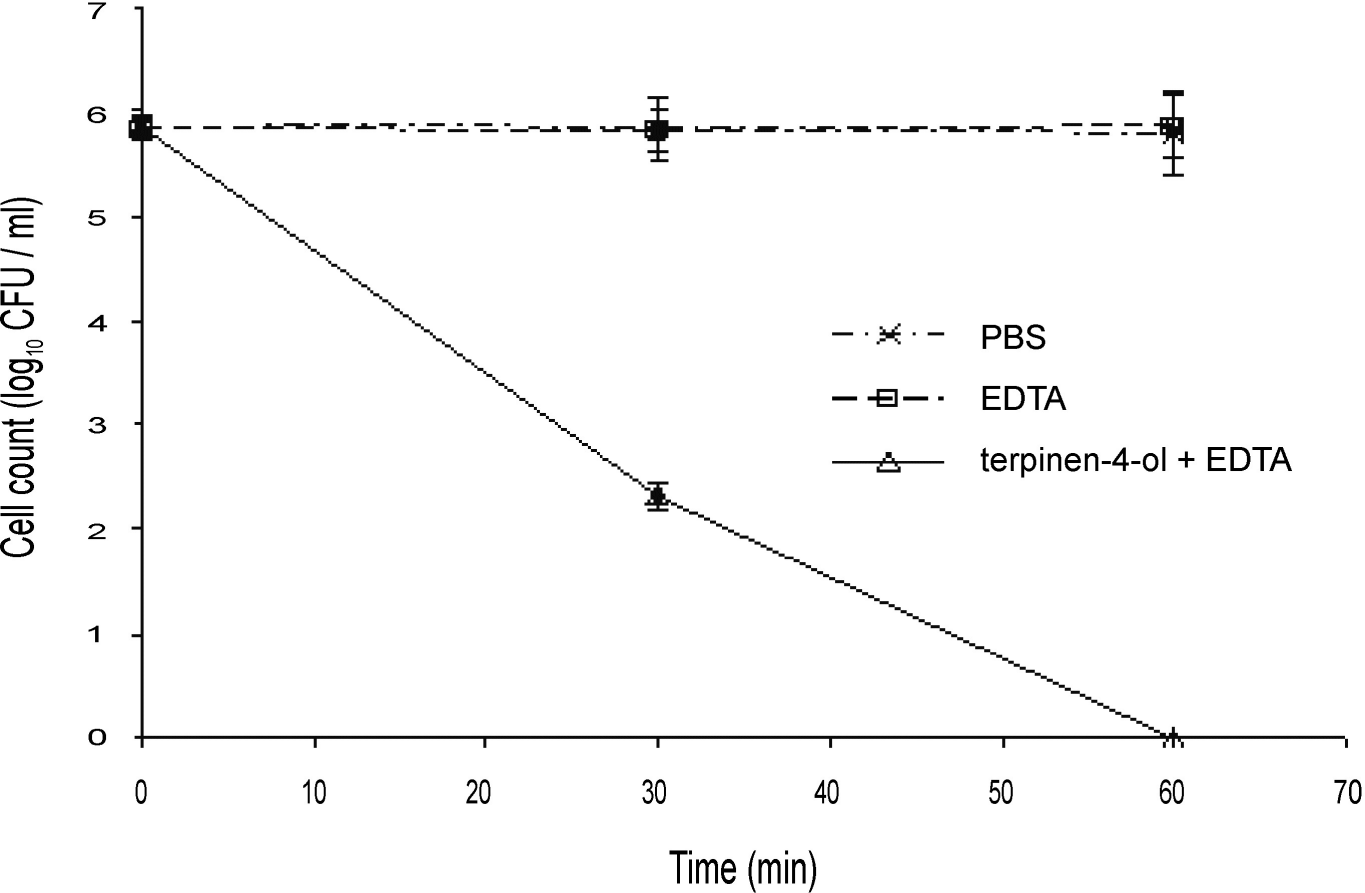
Time-kill analysis of a stationary phase culture of *Burkholderia cenocepacia* strain LMG 16656 when exposed to terpinen-4-ol (0.125%, v/v) and 5 mM EDTA. Cell counts are mean values (n = 6); error bars show standard deviation.

**Fig 6:**
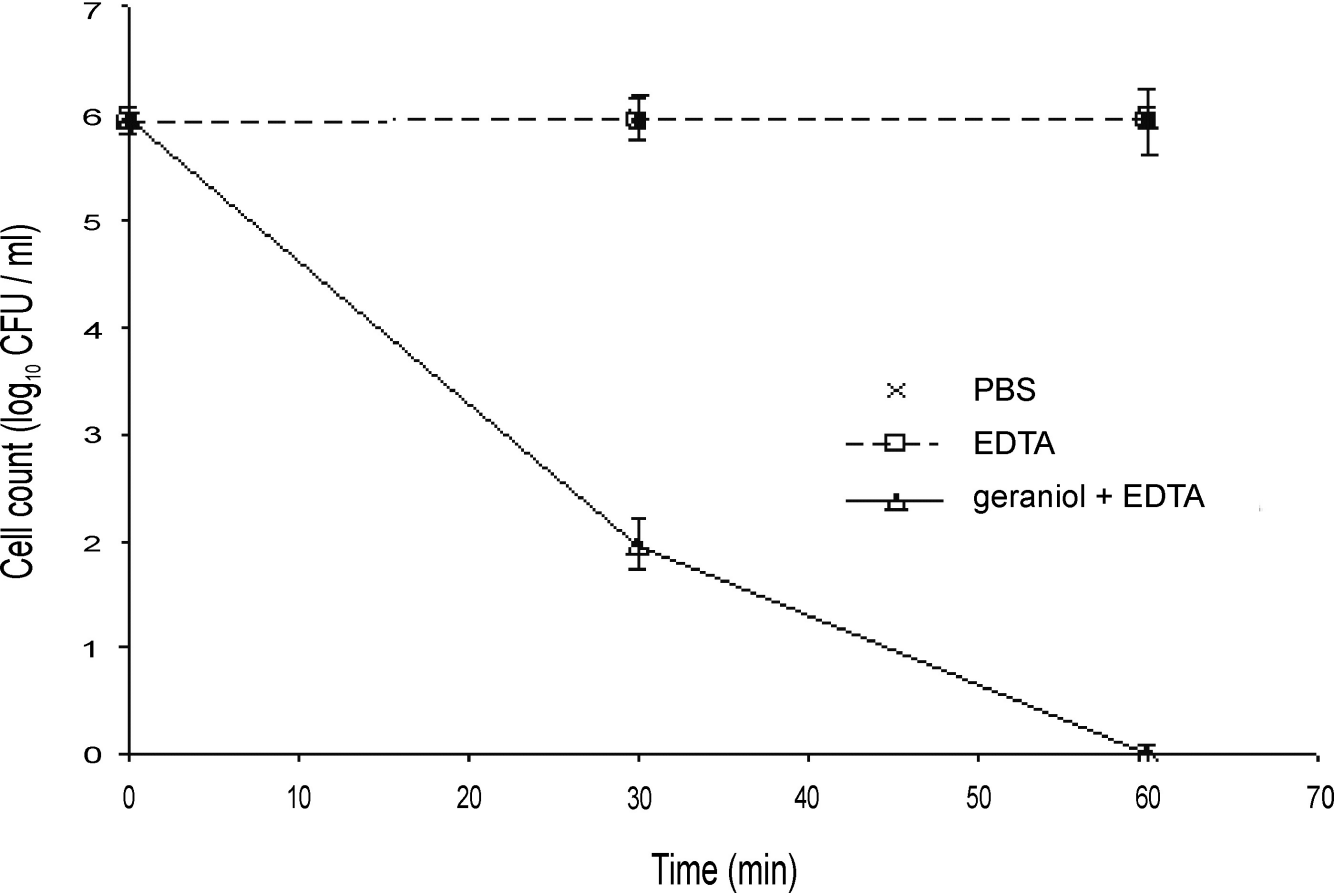
Time-kill analysis of a stationary phase culture of *Burkholderia cenocepacia* strain LMG 16656 when exposed to geraniol (0.25%, v/v) and 5 mM EDTA. Cell counts are mean values (n = 6); error bars show standard deviation.

Several outer membrane permeabilizers such as EDTA have been shown to improve the susceptibility of the resistant pathogen *P*. *aeruginosa* to various antibiotics and plant extracts [39]. The MIC of EDTA against Bcc members was 20 mM and there was no difference in the growth of the Bcc in the presence of 5 mM or 10 mM EDTA. However, while 5 mM EDTA alone had no apparent antimicrobial effect, incorporation of EDTA into susceptibility studies using the broth dilution method reduced the concentration of terpinen-4-ol and geraniol required to kill the Bcc by two-fold, and so these lower concentrations (0.125% and 0.25%, respectively) were used in time kill studies with EDTA (Figs 5 and 6). After 30 min of treatment with either EOC in combination with EDTA, there was at least a 2 log_10_ reduction compared to the cells treated with EOC alone (Figs 1, 2, 5 and 6). In both cases viability with EDTA fell below the detection level by 60 min post exposure. When terpinen-4-ol and geraniol alone were used, 4 h and 3 h, respectively were required for the cell count to fall below the detection level.

### Characterisation of geraniol and terpinen-4-ol mechanisms of action against Bcc using time-kill studies in the presence of an efflux inhibitor

**Fig 7:**
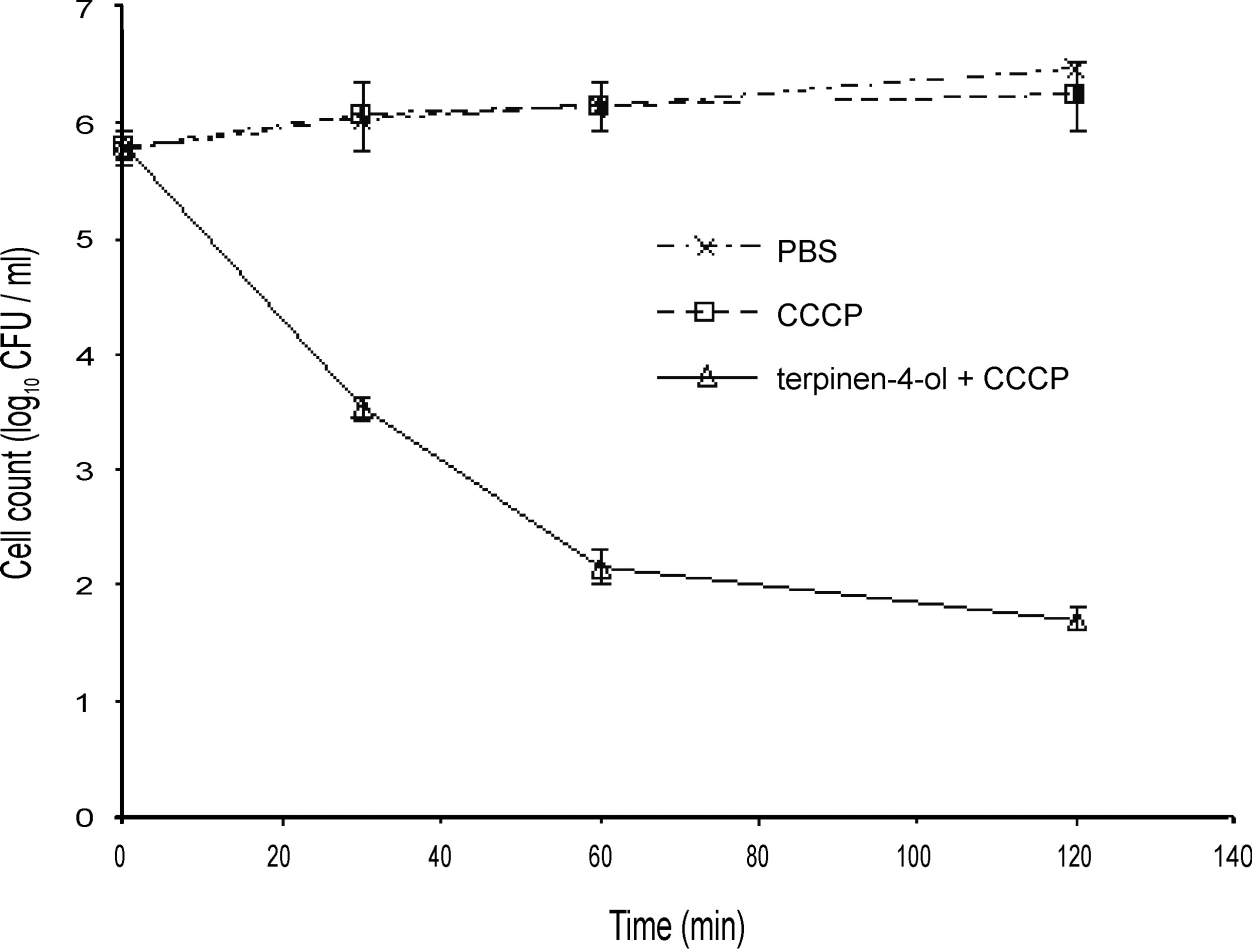
Time-kill analysis of a mid-exponential phase culture of *Burkholderia cenocepacia* strain LMG 16656 when exposed to terpinen-4-ol (0.125%, v/v) and 250 μM CCCP. Cell counts are mean values (n = 6); error bars show standard deviation.

**Fig 8:**
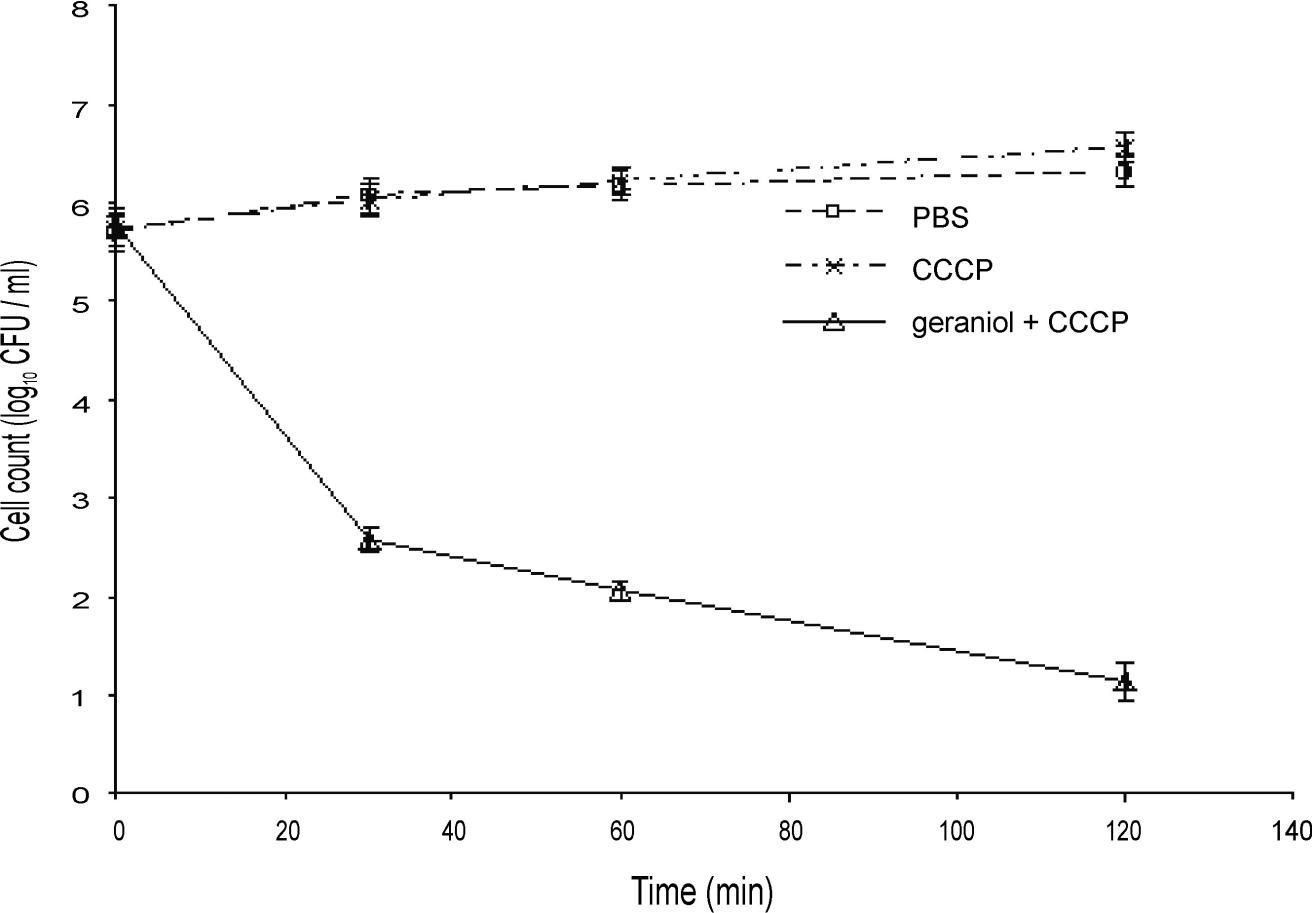
Time-kill analysis of a mid-exponential phase culture of *Burkholderia cenocepacia* strain LMG 16656 when exposed to geraniol (0.25%, v/v) and 250 μM CCCP. Cell counts are mean values (n = 6); error bars show standard deviation.

When energy-dependent efflux mechanisms processes are inhibited, for example by the protonophore carbonyl cyanide m-chlorophenylhydrazone (CCCP), the accumulation and hence activity of antibiotics normally subject to efflux increases [40]. The bactericidal concentration of CCCP against the Bcc was found to be 500 μM and so, to avoid toxicity, testing was carried out at 250 μM. CCCP increased sensitivity to terpinen-4-ol (Fig 7) and geraniol (Fig 8) against the Bcc. Although there was little difference in the reduction in cell numbers, the time required to reach that viable number was reduced in the presence of CCCP (compare Figs 1B, 2B, 7 and 8).

### Loss of cell membrane integrity: Leakage of potassium ions

**Fig 9:**
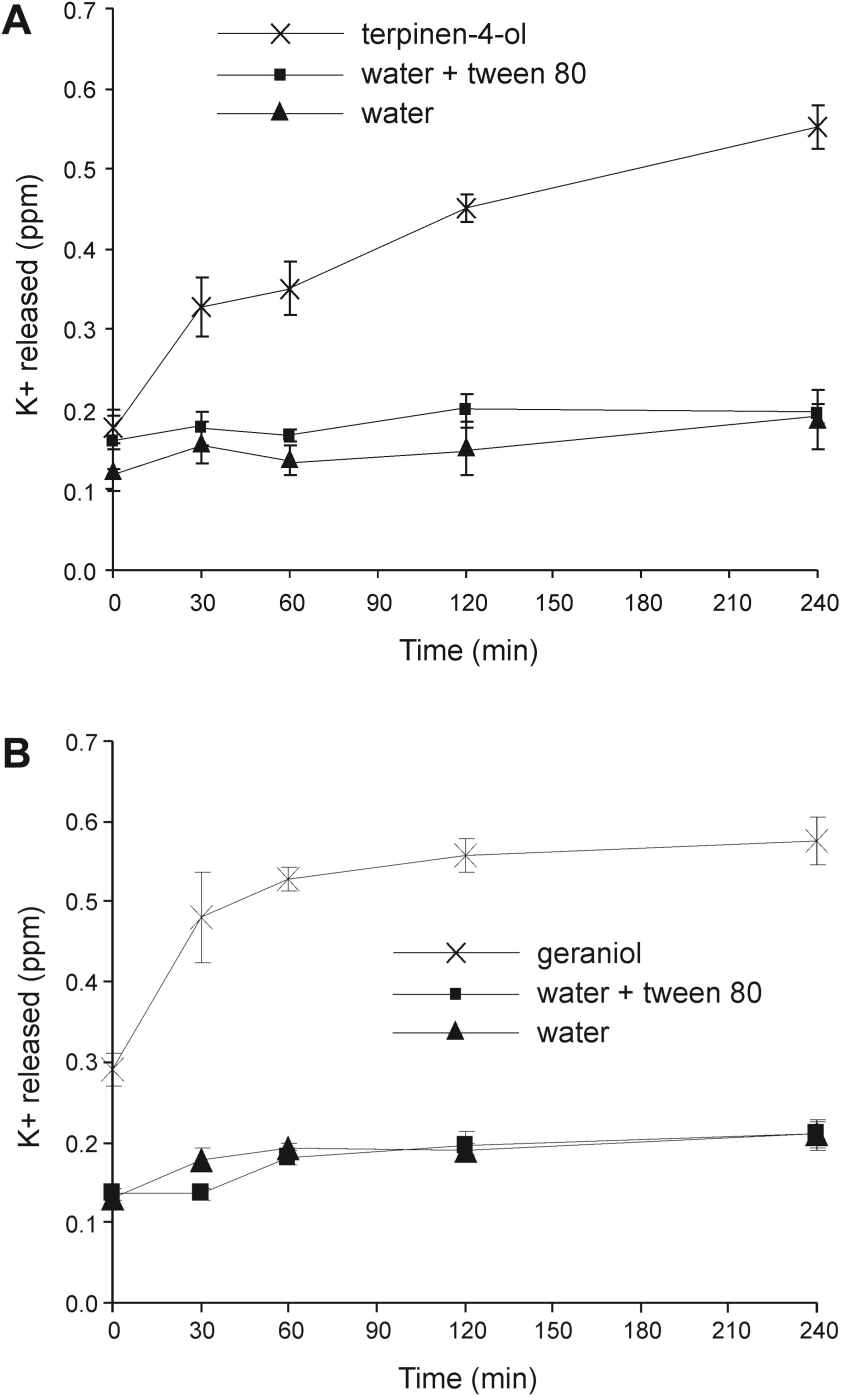
Leakage of potassium from *Burkholderia cenocepacia* strain LMG 16656 when exposed toessential oil components. Cultures were exposed to (A) terpinen-4-ol (0.25%, v/v); (B) geraniol (0.5%, v/v).Values shown are means of three replicates; error bars show standard deviation.

As the internal environment of bacteria is rich in potassium ions, leakage of this ion from cells can be used to monitor membrane damage. The addition of either terpinen-4-ol or geraniol caused a significant increase in potassium leakage from *Burkholderia cenocepacia* cells as shown in Fig 9. Leakage caused by geraniol was rapid, mostly occurring in the first 30 min after addition. Leakage caused by terpinen-4-ol was slower but reached approximately the same level by 240 min. There was no obvious difference between the cells suspended in water and water with Tween80 with regard to the potassium release, indicating that the low concentration of Tween80 used as an emulsifying agent for the EOs and EOCs did not significantly damage cell membranes.

### Loss of cell membrane integrity: Leakage of UV (260 nm)—absorbing material

**Fig 10:**
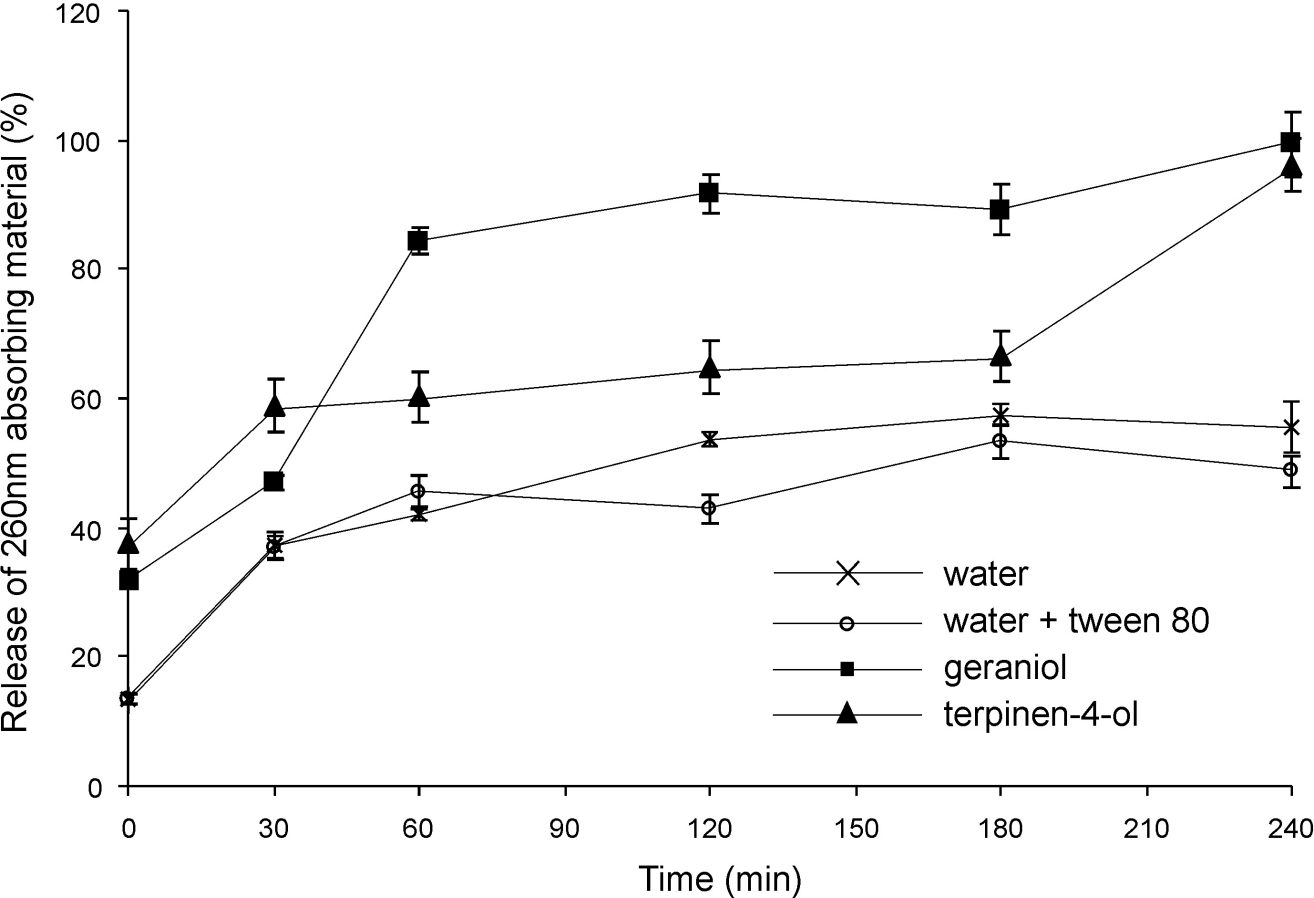
Leakage of 260 nm-absorbing material from 3.2x10^7^ cells of *Burkholderia cenocepacia* strain LMG 16656 when exposed to terpinen-4-ol (0.25%v/v) and geraniol (0.5%v/v). Values shown are normalised to the total material released by heat treatment and means of three replicates; error bars show standard deviation.

If the bacterial membrane is compromised, release of cytoplasmic constituents of the cell such as nucleic acids occurs. Through the detection of absorbance at 260 nm, the amount of nucleic acid content released from the cytoplasm can be measured giving an indication of loss of membrane integrity [41]. Leakage of 260 nm-absorbing material followed essentially the same pattern (Fig 10) as the time-kill studies (Figs 1 and 2). The leakage of macromolecules from *B*. *cenocepacia* after being challenged with the two EOCs showed different patterns, but the total amount of release was approximately the same after 240 min. In the case of geraniol, maximum leakage occurred in the first 60 min, whereas in the case of tepinen-4-ol, gradual release occurred over a period of 180 min and then a rapid increase by 240 min. The cell concentration was 3.2×10^7^ cfu ml^-1^.

### Analysis of membrane fatty acid composition

**Fig 11:**
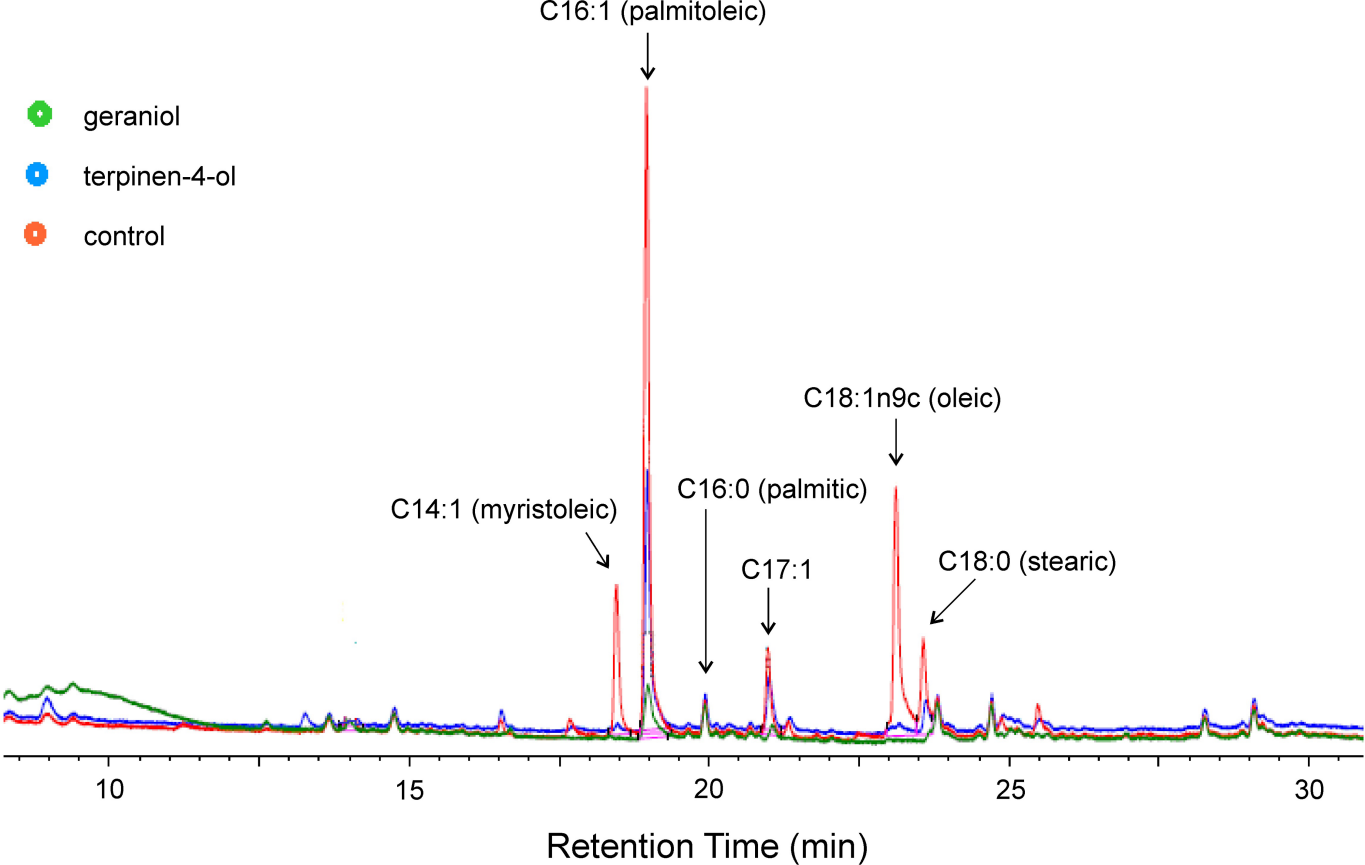
Effect of terpinen-4-ol (0.25%v/v) and geraniol (0.5%v/v) on the membrane fatty acid composition of *Burkholderia cenocepacia* strain LMG 16656. Bacteria were grown at 28°C on TSA. Fatty acid methyl esters (FAME) were analysed by GC.

Alteration of membrane fatty acid profiles of various Gram-negative bacteria in the presence of EOCs have previously been reported [42]. We therefore examined the effect of terpinen-4-ol and geraniol on *B*. *cenocepacia* membrane fatty acid profiles. GC FAME analysis of membrane fatty acids showed clear peaks corresponding to methyl esters of C14:1 (myristoleic), C16:1 (palmitoleic), C16:0 (palmitic), C17:1, C18:0 (stearic) and C18:1n9c (oleic) acids (Fig 11). Clear differences in most of these were observed in the chromatograms obtained for samples treated with terpinen-4-ol or geraniol, specifically C14:1 (myristoleic), C16:1 (palmitoleic), C17:1, C18:0 (stearic) and C18:1n9c (oleic) acids. In each peak area was substantially reduced in either the sample treated with terpinen-4-ol or geraniol or in both treatments.

## Discussion

The 51 Bcc clinical isolates were all typed by MLST to species level and grouped into 18 STs. No novel STs were reported and those isolates provisionally described as ‘other Bcc’ in the collection were most similar to *B*. *cenocepacia*. As expected, STs previously identified as environmental isolates, either within or outside the UK, were identified as causing infection in UK CF patients. This strain collection was felt to be representative of the diversity of strains that might be encountered in patients with very severe CF-related lung disease in the UK during the collection period.

From antibiotic resistance profiles determined for a range of antibiotics, most isolates were clearly multidrug resistant, but there was no obvious association between resistance profiles and STs or species of Bcc. This is consistent with previous studies that have found resistance profiles to be highly heterogeneous through the Bcc and reinforces the need for alternative therapeutic options [12,13,18].

Of the 15 EOs tested, the following six were found to most effective against the Bcc: lavender, lemongrass, marjoram, peppermint, tea tree and rosewood. Of these six, lemongrass and rosewood oils were found to have the highest levels of activity with MIC_50_ and MIC_90_ values of 0.5% and 1.0% respectively. Comparison of MIC and MBC values suggested that all of the 6 oils with significant activity against the Bcc are bacteriocidal in action. A previous agar dilution assay of 47 EOs against a range of Gram-negative rods found these six EOs all to be highly active antimicrobials, with MICs below 2%, but only lemongrass was one of the 3 oils active against *Pseudomonas aeruginosa* [21]. Marjoram EO has been shown to be an effective antimicrobial against *Pseudomonas fluorescens* [23]. A previous assessment of the effects of essential oils on the Bcc tested three of the same oils as the current study and found that tea tree oil was highly effective against 5 out of 18 strains tested; while an EO from a hybrid lavender related to *Lavandula augustifola* was only weakly effective [20]. The same study also found that rosemary oil was one of the most potent anti-Bcc EOs (against 10/18 strains), though we found it to be only moderately effective, perhaps indicating the importance of batch-to-batch variation.

One important caveat about the potential use of EOs as therapeutic drugs is the difficulty in ensuring batch-to-batch control of the component chemicals (EOCs) and their relative concentrations. It may make more sense from this point of view to focus on selected defined EOCs and their potential for anti-Bcc activity, although such an approach may of course lose the advantage of potential antimicrobial synergies between two or more EOCs found in a single oil (reviewed in [43,44]). We have examined a range of EOCs in this study and report that only 2 of them, the alcohols terpinen-4-ol and geraniol, had significant anti-Bcc activity. It is notable when comparing the activities of geraniol and linalool or terpinen-4-ol and α-terpineol, two pairs of highly similar alcohols, that the position of the hydroxyl group seems to be important in determining activity, as previously noted [45]. Geraniol has previously been shown to have a potent antimicrobial activity against members of the *Enterobacteriacae* [24,46] and a rather weak activity against *P*. *aeruginosa* [45,47], while terpinen-4-ol (the major component of tea tree oil) is well known to have broad spectrum antimicrobial properties [47,48,49].

Although several studies have included time kill experiments to assess antibacterial activity of EOs or EOCs, none was focussed on the Bcc [50,51,52,53,54]. Most antibiotic bactericidal activity depends on the bacterial growth phase, often requiring on-going metabolic activity and division of bacterial cells [37]. Nutrient limitation results in the slowing of metabolic processes and the resultant non-growing cells can be highly tolerant to antibiotics [55]. We found that geraniol and terpinen-4-ol were effective against both stationary phase and log phase cultures, although at least in the case of terpinen-4-ol log phase cells were slightly more susceptible. There was no noticeable difference in the decline of mid-exponential phase cells of the Bcc upon challenge with terpinen-4-ol (Fig 3) and geraniol (Fig 4) in PBS compared with the same challenge in ISB (Figs 1 and 2). It is therefore assumed that the cells do not need to be growing for those compounds to exert a bactericidal effect.

As with other Gram negative pathogens, efflux pumps play an important role in multi-drug-resistance in Bcc bacteria and in resistance to antimicrobial chemicals more widely [56,57]. The time-kill experiments reported here suggest that energy-dependent efflux mechanisms are involved in determining the accumulation of terpinen-4-ol or geraniol inside Bcc cells and therefore that it may be possible to increase the anti-Bcc activity of these EOCs using an efflux inhibition strategy.

Many antimicrobial compounds that act on the bacterial cytoplasmic membrane, including chlorhexidine Tea Tree oil and Lemongrass oil, induce the loss of 260 nm-absorbing materials assumed to be nucleic acids [41,58,50]. Potassium leakage is also a useful indicator of membrane damage as it leaks out of the cells very rapidly, and can be easily detected by atomic absorption spectrophotometry [59]. The results from both of these assays strongly suggest that geraniol and terpinen-4-ol are membrane-disrupters. In agreement with this, changes in the membrane fatty acid composition were readily apparent by GC analysis–specifically in the following fatty acids C14:1 (myristoleic), C16:1 (palmitoleic), C17:1, C18:0 (stearic) and C18:1n9c (oleic).

This study began by confirming widespread and heterogenous multidrug resistance in a Bcc clinical isolate collection. Assessment of antimicrobial activity of a range of EOs and EOCs against these Bcc isolates identified six EOs with significant activity and demonstrated that terpinen-4-ol and geraniol were effective anti-Bcc agents. Detailed mechanistic studies suggest that these 2 EOCs are effective against non-growing cells in an efflux dependent manner and that the observed anti-Bcc activity is based on membrane disruption. Whether or not any of these EOs or EOCs ultimately prove useful as therapeutic drugs, for example to treat Bcc infections in CF patients, will depend on a number of factors including pharmacokinetics and toxicology. Geraniol would generally seem to be of very low toxicity and its use in cosmetic fragrances already results in substantial human exposure [60]. Terpinen-4-ol has previously been suggested as a potentially useful topical antimicrobial on the basis of a lack of toxicity against fibroblast cells *in vitro* [61] and incidentally has notable anti-inflammatory properties [37,62].

## Supporting information

S1 Table MICs (mg l-1) of antibiotics against members of the Bcc. (DOCX)

S2 Table MICs (% v/v) and MBCs (% v/v) of six plant essential oils against Bcc strains. (DOCX)

S3 Table Chromatographic profile of marjoram oil. (DOCX)

S4 Table Chromatographic profile of tea tree oil. (DOCX)

S5 Table Chromatographic profile of rosewood oil. (DOCX)

S6 Table Chromatographic profile of lemongrass oil. (DOCX)

S7 Table MICs and MBCs (%v/v) of two essential oil components, terpinen-4-ol and geraniol against clinical isolates and control strains of the Bcc. (DOCX)
