# Supplementary material for: Antimicrobial activity of essential oils against multidrug-resistant clinical isolates of the Burkholderia cepacia complex
Source: PLoS One. 2018 Aug 2;13(8):e0201835. doi: 10.1371/journal.pone.0201835 (PMC6072103; doi:10.1371/journal.pone.0201835)
Supplement: S1 Table — (DOCX) [file pone.0201835.s001.docx]

**S1 Table. MICs (mg l^-1^) of antibiotics against members of the Bcc.**

| **Strain No** | **Genomovar** | **Tobra** | **Cipro** | **Trimeth** | **Mero** | **Colis** | **Tet** | **Ceftaz** | **Aztreo** |
| --- | --- | --- | --- | --- | --- | --- | --- | --- | --- |
| 314270 | *B. cenocepacia* IIIB | >256 | >256 | 2 | 1 | >512 | >256 | 2 | 16 |
| 37463 | *B. cenocepacia* IIIB | 256 | 256 | 1 | 4 | >512 | 8 | 32 | 64 |
| 322107Q | *B. cenocepacia* IIIA | 128 | 32 | 2 | 8 | >512 | 8 | 64 | >256 |
| 344398L | Other Bcc | >256 | 32 | 4 | 4 | >512 | 64 | 64 | >256 |
| 35164 | *B. vietnamiensis* | 256 | 16 | 8 | 64 | >512 | 64 | 256 | >256 |
| 356892Q | *B. cenocepacia* IIIB | >256 | 128 | 128 | 8 | >512 | 64 | 8 | >256 |
| 344958 | Other Bcc | 32 | 128 | 0.5 | 8 | >512 | 64 | 4 | 8 |
| 330658J | *B. dolasa* | 64 | 64 | 32 | 64 | >512 | 8 | 32 | >256 |
| 334756 | Other Bcc | 32 | 1 | 1 | 1 | >512 | 16 | 2 | 8 |
| 333874 | *B. cenocepacia* IIIB | >256 | >256 | 8 | 16 | >512 | 16 | 128 | >256 |
| 0030831E | *B. cenocepacia* IIIA | >256 | 32 | 16 | 16 | >512 | 64 | 64 | >256 |
| ME000947Z | *B. contaminans* | 128 | 2 | 2 | 8 | >512 | 8 | 4 | 256 |
| M9925 | *B. cepacia* | >256 | 4 | 8 | 8 | >512 | 32 | 32 | 128 |
| 379392 | *B. vietnamiensis* | 64 | 256 | 16 | 4 | >512 | 2 | 128 | 128 |
| 495598D | *B. cenocepacia* IIIA | 64 | 4 | 2 | 0.5 | >512 | 2 | 2 | 2 |
| 498829 | *B. cenocepacia* IIIB | 64 | 8 | 128 | 8 | >512 | 8 | 32 | 128 |
| MR15953 | *B. vietnamiensis* | 128 | 4 | 1 | 1 | >512 | 2 | 4 | 4 |
| 501869W | *B. multivorans* | >256 | >256 | 2 | 4 | >512 | 128 | 32 | 256 |
| 536766T | *B. cenocepacia* IIIB | >256 | >256 | 1 | 4 | >512 | 64 | 32 | 256 |
| 537524 | Other Bcc | 64 | 256 | 256 | 32 | >512 | 1 | 8 | 2 |
| 565350 | *B. multivorans* | 64 | 8 | 2 | 1 | >512 | 8 | 32 | 64 |
| 518064-61 | Other Bcc | 128 | 8 | 2 | 16 | >512 | 2 | >256 | 128 |
| 518064-62 | Other Bcc | 256 | 8 | 0.5 | 1 | >512 | 32 | 2 | 4 |
| 537607 | Other Bcc | >256 | >256 | 64 | 2 | >512 | 32 | 64 | 128 |
| 553728 | Other Bcc | >256 | 256 | 8 | 4 | >512 | 16 | >256 | >256 |
| 556478 | Other Bcc | 128 | 4 | 2 | 2 | >512 | 8 | 32 | 256 |
| 562964 | *B. ambifaria* | 64 | 16 | 128 | 4 | >512 | 64 | 8 | 32 |
| 565708X | *B. cenocepacia* IIIA | 128 | 8 | 1 | 2 | >512 | 32 | 2 | 4 |
| 579415 | *B. cenocepacia* IIIA | >256 | >256 | 8 | 4 | >512 | 128 | 128 | >256 |
| H0298-0221 | *B. ambifaria* | >256 | >256 | 16 | 8 | >512 | 16 | 128 | >256 |
| H06036-0378 | *B. vietnamiensis* | 64 | 16 | 2 | 0.5 | >512 | 4 | 8 | 4 |
| 539117 | *B. cenocepacia* IIID | 64 | 32 | 8 | 16 | >512 |  | 128 | >256 |
| 601615 | Other Bcc | 128 | 4 | 8 | 0.5 | >512 | 4 | 4 | 4 |
| 611313 | Other Bcc | 64 | 32 | 8 | 4 | >512 | 64 | 4 | 32 |
| MR23273 | *B. vietnamiensis* | 64 | 4 | 1 | 1 | >512 | 2 | 4 | 4 |
| BCH95-26284 | *B. ambifaria* | 128 | >256 | 128 | 8 | >512 | 16 | 4 | 32 |
| 674880Y | *B. cenocepacia* IIIA | 64 | 4 | 16 | <0.06 | >512 | 16 | 1 | 0.25 |
| 666160 | *B. cenocepacia* IIIA | 64 | 2 | 1 | 1 | >512 | 16 | 4 | 4 |
| 680045 | *B. cenocepacia* IIIB | 8 | 4 | 16 | 4 | >512 | 8 | 32 | 0.5 |
| 642190 | *B. dolasa* | 64 | 256 | 32 | 8 | >512 | 16 | 32 | 64 |
| 666432 | *B. vietnamiensis* | 128 | 128 | 16 | 8 | >512 | 64 | 1 | 32 |
| 430797 | *B. anthina* | 64 | 256 | 16 | 8 | >512 | 2 | 1 | 4 |
| ?3124 | Other Bcc | >256 | 64 | 4 | 8 | >512 | 64 | 2 | 64 |
| 491988L | *B. cenocepacia* IIIB | 64 | 256 | 64 | 2 | >512 | 2 | 16 | 1 |
| 325658S | *B. cenocepacia* IIIA | 64 | 32 | 2 | 8 | >512 | 16 | 64 | >256 |
| 19 | *B. dolasa* | >256 | 64 | 8 | 64 | >512 | 64 | 128 | >256 |
| 367323 | *B. cenocepacia* IIIA | 64 | 2 | 4 | 2 | >512 | 8 | 16 | 4 |
| 1RJ | *B. multivorans* | >256 | 64 | 8 | 128 | >512 | 64 | 128 | >256 |
| 552264 | *B. cenocepacia* IIIB | >256 | 32 | 1 | 16 | >512 | 16 | 64 | >256 |
| 566570 | *B. cenocepacia* IIIB | >256 | 256 | 128 | 16 | >512 | 8 | 128 | >256 |
| 53 | *B. vietnamiensis* | 64 | 2 | 1 | 1 | >512 | 4 | 8 | 2 |
| LMG 16656 | *B. cenocepacia* IIIA | 64 | 1 | 0.5 | 0.5 | >512 | 32 | 16 | 1 |
| NCTC 10662 | *Pseudomonas aeruginosa* | 0.5 | 0.25 | 32 | 0.125 | 2 | 16 | 1 | 2 |

Tobra, tobramycin; Cipro, ciprofloxacin; Trimeth, trimethoprim; Mero, meropenem; Colis, colistin; Tet, tetracycline; Ceftaz, ceftazidime; Aztreo, aztreonam.
