# Supplementary material for: Antimicrobial activity of essential oils against multidrug-resistant clinical isolates of the Burkholderia cepacia complex
Source: PLoS One. 2018 Aug 2;13(8):e0201835. doi: 10.1371/journal.pone.0201835 (PMC6072103; doi:10.1371/journal.pone.0201835)
Supplement: S2 Table — (DOCX) [file pone.0201835.s002.docx]

**S2 Table. MICs (% v/v) and MBCs (% v/v) of six plant essential oils against Bcc strains. Values are means of three replicates.**

| **_Strain No_** | **_Lemongrass_** | | **_Marjoram_** | | **_Teatree_** | | **_Rosewood_** | | **_Lavender_** | | **_Peppermint_** | |
| --- | --- | --- | --- | --- | --- | --- | --- | --- | --- | --- | --- | --- |
|  | **_MIC_** | **_MBC_** | **_MIC_** | **_MBC_** | **_MIC_** | **_MBC_** | **_MIC_** | **_MBC_** | **_MIC_** | **_MBC_** | **_MIC_** | **_MBC_** |
| _312207Q_ | _0.5_ | _8_ | _2_ | _2_ | _2_ | _4_ | _1_ | _4_ | _2_ | _>8_ | _2_ | _>8_ |
| _0030831E_ | _0.5_ | _1_ | _2_ | _2_ | _2_ | _2_ | _0.5_ | _0.5_ | _2_ | _8_ | _1_ | _4_ |
| _495598D_ | _1_ | _1_ | _4_ | _2_ | _2_ | _4_ | _0.5_ | _0.5_ | _2_ | _>8_ | _1_ | _8_ |
| _565708X_ | _0.5_ | _1_ | _2_ | _2_ | _2_ | _4_ | _0.125_ | _0.125_ | _1_ | _8_ | _0.5_ | _4_ |
| _579415_ | _1_ | _4_ | _4_ | _4_ | _2_ | _4_ | _1_ | _2_ | _2_ | _>8_ | _2_ | _8_ |
| _674880Y_ | _1_ | _8_ | _4_ | _4_ | _4_ | _8_ | _1_ | _2_ | _1_ | _4_ | _2_ | _>8_ |
| _666160_ | _1_ | _4_ | _2_ | _2_ | _1_ | _2_ | _0.5_ | _2_ | _1_ | _>8_ | _0.5_ | _4_ |
| _325658S_ | _0.5_ | _1_ | _1_ | _1_ | _4_ | _8_ | _2_ | _2_ | _2_ | _>8_ | _2_ | _>8_ |
| _367323_ | _1_ | _8_ | _2_ | _4_ | _4_ | _8_ | _2_ | _2_ | _2_ | _8_ | _1_ | _8_ |
| _566570_ | _0.5_ | _0.5_ | _1_ | _4_ | _2_ | _4_ | _1_ | _4_ | _2_ | _>8_ | _1_ | _>8_ |
| _37463_ | _1_ | _4_ | _2_ | _4_ | _2_ | _2_ | _1_ | _4_ | _1_ | _>8_ | _1_ | _4_ |
| _356892Q_ | _0.5_ | _1_ | _2_ | _2_ | _2_ | _4_ | _1_ | _2_ | _2_ | _>8_ | _2_ | _>8_ |
| _333874_ | _1_ | _4_ | _4_ | _4_ | _2_ | _4_ | _0.5_ | _0.5_ | _1_ | _8_ | _2_ | _4_ |
| _536766T_ | _0.5_ | _1_ | _1_ | _1_ | _2_ | _2_ | _0.5_ | _2_ | _1_ | _>8_ | _2_ | _>8_ |
| _491988L_ | _1_ | _2_ | _2_ | _2_ | _1_ | _2_ | _0.125_ | _1_ | _2_ | _>8_ | _2_ | _>8_ |
| _552264_ | _0.5_ | _4_ | _1_ | _8_ | _2_ | _4_ | _0.5_ | _4_ | _0.5_ | _>8_ | _1_ | _4_ |
| _539117_ | _0.5_ | _2_ | _2_ | _2_ | _1_ | _4_ | _0.5_ | _2_ | _2_ | _8_ | _2_ | _>8_ |
| _314270_ | _1_ | _4_ | _4_ | _4_ | _2_ | _4_ | _0.125_ | _0.5_ | _2_ | _>8_ | _2_ | _8_ |
| _498829_ | _1_ | _4_ | _2_ | _4_ | _2_ | _4_ | _1_ | _1_ | _2_ | _>8_ | _2_ | _>8_ |
| _680045_ | _1_ | _4_ | _2_ | _4_ | _4_ | _4_ | _0.5_ | _2_ | _1_ | _>8_ | _2_ | _>8_ |
| _M9925_ | _0.5_ | _1_ | _1_ | _2_ | _1_ | _2_ | _1_ | _1_ | _2_ | _>8_ | _2_ | _>8_ |
| _35164_ | _0.5_ | _2_ | _4_ | _4_ | _4_ | _8_ | _0.5_ | _1_ | _2_ | _>8_ | _2_ | _>8_ |
| _379392_ | _1_ | _4_ | _4_ | _4_ | _2_ | _8_ | _0.5_ | _4_ | _2_ | _>8_ | _2_ | _>8_ |
| _MR15953_ | _0.5_ | _1_ | _2_ | _4_ | _2_ | _2_ | _0.5_ | _2_ | _2_ | _>8_ | _0.5_ | _4_ |
| _H06036-0378_ | _1_ | _4_ | _1_ | _2_ | _2_ | _4_ | _1_ | _2_ | _2_ | _>8_ | _2_ | _>8_ |
| _MR23273_ | _1_ | _2_ | _2_ | _4_ | _2_ | _4_ | _0.5_ | _2_ | _1_ | _>8_ | _2_ | _>8_ |
| _666432_ | _0.5_ | _2_ | _1_ | _4_ | _1_ | _2_ | _1_ | _2_ | _1_ | _>8_ | _2_ | _>8_ |
| _53_ | _1_ | _4_ | _2_ | _4_ | _2_ | _4_ | _0.125_ | _0.5_ | _2_ | _>8_ | _2_ | _8_ |
| _19_ | _1_ | _4_ | _2_ | _4_ | _2_ | _4_ | _0.125_ | _0.5_ | _2_ | _>8_ | _2_ | _>8_ |
| _642190_ | _1_ | _4_ | _1_ | _2_ | _2_ | _4_ | _1_ | _4_ | _1_ | _>8_ | _2_ | _>8_ |
| _330658J_ | _0.125_ | _0.125_ | _2_ | _4_ | _2_ | _8_ | _0.125_ | _0.125_ | _2_ | _>8_ | _1_ | _4_ |
| _501869W_ | _1_ | _4_ | _1_ | _1_ | _2_ | _4_ | _0.5_ | _2_ | _1_ | _8_ | _1_ | _8_ |
| _1RJ_ | _0.5_ | _2_ | _4_ | _4_ | _4_ | _4_ | _0.125_ | _0.125_ | _1_ | _>8_ | _0.5_ | _8_ |
| _565350_ | _0.5_ | _8_ | _1_ | _2_ | _2_ | _4_ | _0.5_ | _4_ | _1_ | _>8_ | _1_ | _4_ |
| _430797_ | _0.5_ | _8_ | _2_ | _4_ | _2_ | _8_ | _0.5_ | _0.5_ | _2_ | _>8_ | _2_ | _>8_ |
| _H0298-0221_ | _1_ | _4_ | _2_ | _2_ | _2_ | _4_ | _0.125_ | _0.5_ | _2_ | _>8_ | _0.5_ | _4_ |
| _BCH95-26284_ | _0.5_ | _0.5_ | _1_ | _2_ | _1_ | _4_ | _0.5_ | _1_ | _2_ | _>8_ | _1_ | _4_ |
| _562964_ | _0.5_ | _2_ | _2_ | _2_ | _2_ | _4_ | _0.5_ | _2_ | _2_ | _>8_ | _2_ | _>8_ |
| _518064_ | _0.5_ | _1_ | _2_ | _4_ | _2_ | _4_ | _0.5_ | _2_ | _2_ | _>8_ | _2_ | _>8_ |
| _518064_ | _1_ | _4_ | _2_ | _4_ | _2_ | _4_ | _0.125_ | _0.125_ | _1_ | _>8_ | _2_ | _>8_ |
| _556478_ | _0.5_ | _1_ | _1_ | _2_ | _2_ | _2_ | _0.5_ | _4_ | _0.5_ | _4_ | _2_ | _4_ |
| _601615_ | _0.5_ | _2_ | _1_ | _1_ | _2_ | _8_ | _0.125_ | _2_ | _2_ | _>8_ | _2_ | _>8_ |
| _?3124_ | _0.5_ | _1_ | _1_ | _1_ | _2_ | _2_ | _1_ | _4_ | _2_ | _>8_ | _1_ | _4_ |
| _537607_ | _1_ | _4_ | _4_ | _4_ | _4_ | _4_ | _1_ | _1_ | _1_ | _8_ | _0.5_ | _4_ |
| _553728_ | _1_ | _4_ | _2_ | _2_ | _1_ | _2_ | _1_ | _4_ | _2_ | _>8_ | _2_ | _>8_ |
| _611313_ | _0.5_ | _1_ | _2_ | _2_ | _2_ | _4_ | _0.5_ | _1_ | _2_ | _>8_ | _2_ | _>8_ |
| _344958_ | _1_ | _4_ | _2_ | _4_ | _2_ | _4_ | _1_ | _4_ | _2_ | _>8_ | _2_ | _>8_ |
| _334756_ | _0.5_ | _0.5_ | _2_ | _2_ | _2_ | _4_ | _0.5_ | _2_ | _2_ | _>8_ | _1_ | _>8_ |
| _537524_ | _0.5_ | _0.5_ | _1_ | _1_ | _1_ | _1_ | _0.125_ | _0.125_ | _1_ | _8_ | _1_ | _8_ |
| _344398L_ | _1_ | _8_ | _1_ | _8_ | _1_ | _4_ | _0.5_ | _4_ | _1_ | _>8_ | _0.5_ | _4_ |
| _ME000947Z_ | _1_ | _4_ | _4_ | _4_ | _2_ | _8_ | _1_ | _4_ | _2_ | _>8_ | _2_ | _>8_ |
| _16232_ | _1_ | _4_ | _2_ | _2_ | _2_ | _4_ | _0.5_ | _1_ | _2_ | _>8_ | _1_ | _8_ |
| _18863_ | _0.5_ | _1_ | _2_ | _4_ | _2_ | _8_ | _0.5_ | _1_ | _1_ | _8_ | _1_ | _4_ |
| _18826_ | _0.5_ | _1_ | _1_ | _2_ | _1_ | _2_ | _0.5_ | _1_ | _2_ | _>8_ | _2_ | _>8_ |
| _18825_ | _0.5_ | _0.5_ | _2_ | _4_ | _2_ | _4_ | _1_ | _2_ | _2_ | _>8_ | _2_ | _>8_ |
| _18870_ | _1_ | _4_ | _2_ | _2_ | _2_ | _4_ | _0.125_ | _0.5_ | _2_ | _>8_ | _2_ | _>8_ |
| _17997_ | _1_ | _2_ | _1_ | _2_ | _2_ | _4_ | _0.5_ | _1_ | _2_ | _8_ | _2_ | _8_ |
