# Supplementary material for: Antimicrobial activity of essential oils against multidrug-resistant clinical isolates of the Burkholderia cepacia complex
Source: PLoS One. 2018 Aug 2;13(8):e0201835. doi: 10.1371/journal.pone.0201835 (PMC6072103; doi:10.1371/journal.pone.0201835)
Supplement: S3 Table — (DOCX) [file pone.0201835.s003.docx]

**S3 Table. Chromatographic profile of marjoram oil**

| **Peak** | **Retention time (min)** | **SI^a^** | **RSI^b^** | **Library identification** | **Present in**  **ISO 4728:2003** |
| --- | --- | --- | --- | --- | --- |
| 1 | 4.78 | 943 | 943 | α- pinene | Yes |
| 2 | 5.08 | 932 | 936 | camphene | Yes |
| 3 | 5.70 | 946 | 946 | β- pinene | Yes |
| 4 | 6.01 | 883 | 883 | β-myrcene | Yes |
| 5 | 6.91 | 916 | 916 | p-cymene | Yes |
| 6 | 7.14 | 888 | 888 | eucalyptol | Yes |
| 7 | 7.68 | 911 | 911 | γ-terpinene |  |
| 8 | 8.74-8.94 | 914 | 917 | linalool | Yes |
| 9 | 10.01 | 965 | 965 | D-camphor | Yes |
| 10 | 10.31 | 947 | 947 | borneol | Yes |
| 11 | 11.23 | 953 | 953 | δ-terpineol | Yes |
| 12 | 11.43 | 881 | 881 | terpinen-4-ol | Yes |
| 13 | 12.90 | 957 | 957 | linalyl acetate | Yes |
| 14 | 15.37 | 918 | 918 | α-terpinyl acetate |  |
| 15 | 17.11 | 936 | 936 | β-caryophyllene | Yes |

a) Similarity index

b) Reverse similarity index
