# Supplementary material for: Antimicrobial activity of essential oils against multidrug-resistant clinical isolates of the Burkholderia cepacia complex
Source: PLoS One. 2018 Aug 2;13(8):e0201835. doi: 10.1371/journal.pone.0201835 (PMC6072103; doi:10.1371/journal.pone.0201835)
Supplement: S4 Table — (DOCX) [file pone.0201835.s004.docx]

**S4 Table. Chromatographic profile of tea tree oil**

| **Peak** | **Retention time (min)** | **SI^a^** | **RSI^b^** | **Library identification** | **Present in**  **ISO 4720:2004** |
| --- | --- | --- | --- | --- | --- |
| 1 | 4.85 | 933 | 933 | α-thujene | Yes |
| 2 | 5.01 | 957 | 953 | α-pinene | Yes |
| 3 | 5.86 | 941 | 945 | sabinene | Yes |
| 4 | 5.94 | 909 | 909 | β-pinene | Yes |
| 5 | 6.24 | 889 | 901 | myrcene | Yes |
| 6 | 6.96 | 921 | 918 | α-terpinene | Yes |
| 7 | 7.12 | 901 | 913 | p-cymene | Yes |
| 8 | 7.30 | 889 | 887 | 1,8-cineole | Yes |
| 9 | 8.11 | 946 | 936 | Γ-terpinene | Yes |
| 10 | 8.77 | 907 | 911 | terpinolene | Yes |
| 11 | 11.54 | 936 | 936 | terpinen-4-ol | Yes |
| 12 | 11.72 | 965 | 958 | α-terpineol | Yes |
| 13 | 17.89 | 914 | 914 | aromadendrene | Yes |
| 14 | 19.30 | 918 | 918 | ledene | Yes |
| 15 | 19.96 | 889 | 901 | Δ-cadinene | Yes |
| 16 | 21.39 | 903 | 915 | globulol | Yes |

a) Similarity index

b) Reverse similarity index
