# Supplementary material for: Antimicrobial activity of essential oils against multidrug-resistant clinical isolates of the Burkholderia cepacia complex
Source: PLoS One. 2018 Aug 2;13(8):e0201835. doi: 10.1371/journal.pone.0201835 (PMC6072103; doi:10.1371/journal.pone.0201835)
Supplement: S5 Table — (DOCX) [file pone.0201835.s005.docx]

**S5 Table. Chromatographic profile of rosewood oil.**

| **Peak** | **Retention time (min)** | **SI** | **RSI** | **Library identification** | **Present in**  **ISO 3761:2005** |
| --- | --- | --- | --- | --- | --- |
| 1 | 4.99 | 899 | 906 | α-pinene | Yes |
| 2 | 5.80 | 884 | 903 | geranic oxide | Yes |
| 3 | 7.15 | 916 | 920 | D-limonene |  |
| 4 | 8.34 | 909 | 910 | linalool oxide | Yes |
| 5 | 9.61 | 908 | 908 | β-linalool | Yes |
| 6 | 10.07 | 778 | 787 | 1,2-dihydrolinalool |  |
| 7 | 11.63 | 896 | 897 | α-terpineol | Yes |
| 8 | 13.24 | 915 | 917 | citrol | Yes |
| 9 | 16.26 | 909 | 912 | ylangene |  |
| 10 | 19.04 | 933 | 939 | β-selinene |  |
| 11 | 19.25 | 896 | 903 | 2-isopropenyl-4a,8-dimethyl-  1,2,3,4,4a,5,6,7-  octahydronaphthalene |  |
| 12 | 25.42 | 910 | 914 | benzyl benzoate | Yes |

a) Similarity index

b) Reverse similarity index
