# Supplementary material for: Antimicrobial activity of essential oils against multidrug-resistant clinical isolates of the Burkholderia cepacia complex
Source: PLoS One. 2018 Aug 2;13(8):e0201835. doi: 10.1371/journal.pone.0201835 (PMC6072103; doi:10.1371/journal.pone.0201835)
Supplement: S6 Table — (DOCX) [file pone.0201835.s006.docx]

**S6 Table. Chromatographic profile of lemongrass oil.**

| **Peak** | **Retention time (min)** | **SI^a^** | **RSI^b^** | **Library identification** | **Present in**  **ISO 4718:2004** |
| --- | --- | --- | --- | --- | --- |
| 1 | 4.72 | 935 | 926 | tricyclene |  |
| 2 | 4.95 | 782 | 922 | α-pinene |  |
| 3 | 5.27 | 934 | 946 | camphene |  |
| 4 | 6.18 | 933 | 939 | 6-methyl-5-heptene-2-one | Yes |
| 5 | 7.12 | 884 | 885 | D-limonene | Yes |
| 6 | 7.34 | 920 | 933 | 2-norpinene,3,6,6,-trimethyl- |  |
| 7 | 7.60 | 884 | 907 | Β-ocimene |  |
| 8 | 8.26 | 905 | 905 | 4-nonanone |  |
| 9 | 8.99 | 897 | 902 | β-linalool |  |
| 10 | 10.35 | 744 | 855 | 2,2-dimethyl-3,4-octadienal |  |
| 11 | 10.42 | 945 | 947 | citronellal |  |
| 12 | 11.25 | 828 | 829 | cis-verbenol |  |
| 13 | 13.19 | 899 | 899 | β-citral | Yes |
| 14 | 13.69 | 866 | 873 | citrol | Yes |
| 15 | 14.12 | 917 | 917 | α-citral | Yes |
| 16 | 16.61 | 902 | 903 | geranyl acetate | Yes |
| 17 | 17.45 | 940 | 941 | caryophyllene | Yes |
| 18 | 19.73 | 893 | 899 | muurolene |  |
| 19 | 19.93 | 865 | 893 | δ-cadinene |  |
| 20 | 21.38 | 896 | 902 | caryophyllene oxide |  |

a) Similarity index

b) Reverse similarity index
