# Supplementary material for: Antimicrobial activity of essential oils against multidrug-resistant clinical isolates of the Burkholderia cepacia complex
Source: PLoS One. 2018 Aug 2;13(8):e0201835. doi: 10.1371/journal.pone.0201835 (PMC6072103; doi:10.1371/journal.pone.0201835)
Supplement: S7 Table — (DOCX) [file pone.0201835.s007.docx]

**S7 Table. The MICs and MBCs (%v/v) of two essential oil components, terpinen-4-ol and geraniol against clinical isolates and control strains of the Bcc.**

| **Strain number** | **Terpinen-4-ol (%v/v)** | | **Geraniol (%v/v)** | |
| --- | --- | --- | --- | --- |
|  | **MIC** | **MBC** | **MIC** | **MBC** |
| 536766T | 0.25 | 0.25 | 0.5 | 0.5 |
| 58(501869W) | 0.25 | 0.25 | 0.5 | 0.5 |
| 518064 | 0.25 | 0.25 | 0.125 | 0.125 |
| MR15953 | 0.25 | 0.25 | 0.5 | 0.5 |
| 601615 | 0.5 | 0.5 | 0.125 | 0.125 |
| 37463 | 0.125 | 0.25 | 0.5 | 0.5 |
| 379392 | 0.5 | 0.5 | 0.5 | 0.5 |
| 322107Q | 0.25 | 0.25 | 1 | 1 |
| 642190 | 0.25 | 0.25 | 1 | 1 |
| 680045 | 0.5 | 0.5 | 0.5 | 0.5 |
| MR23273 | 0.25 | 0.25 | 0.5 | 0.5 |
| 495598D | 0.25 | 0.25 | 0.25 | 0.25 |
| 498829 | 0.25 | 0.25 | 1 | 1 |
| Me0009472 | 0.5 | 0.5 | 1 | 1 |
| 553728 | 0.25 | 0.25 | 1 | 1 |
| 518064(61) | 0.25 | 0.25 | 0.25 | 0.5 |
| 1RJ | 0.5 | 0.5 | 0.125 | 0.125 |
| 14(325658) | 0.5 | 0.5 | 1 | 1 |
| 491988 | 0.25 | 0.25 | 0.125 | 0.25 |
| 344958 | 0.5 | 0.5 | 0.5 | 0.5 |
| 552264 | 0.5 | 0.5 | 0.5 | 0.5 |
| 556478 | 0.5 | 0.5 | 0.25 | 0.25 |
| H0686-0398 | 0.5 | 0.5 | 0.5 | 0.5 |
| 430797 | 0.5 | 0.5 | 0.5 | 0.5 |
| 537524 | 0.125 | 0.125 | 0.125 | 0.125 |
| 19 | 0.5 | 0.5 | 0.125 | 0.25 |
| 344398L | 0.25 | 0.25 | 0.5 | 0.5 |
| 537607 | 0.5 | 0.5 | 1 | 1 |
| 3124 | 0.25 | 0.5 | 0.5 | 0.5 |
| 666160 | 0.25 | 0.25 | 0.5 | 0.5 |
| 0030831E | 0.5 | 0.5 | 0.5 | 0.5 |
| 611313 | 0.5 | 0.5 | 0.5 | 0.5 |
| 674880 | 0.5 | 0.5 | 0.5 | 0.5 |
| 356892 | 0.5 | 0.5 | 1 | 1 |
| BCH95-26284 | 0.25 | 0.5 | 0.5 | 0.5 |
| 367323 | 0.5 | 0.5 | 1 | 1 |
| 333874 | 0.25 | 0.25 | 0.5 | 0.5 |
| 579415 | 0.25 | 0.25 | 0.5 | 0.5 |
| 39(35164) | 0.5 | 0.5 | 0.125 | 0.5 |
| 566570 | 0.25 | 0.25 | 0.5 | 1 |
| 314270 | 0.25 | 0.5 | 0.125 | 0.125 |
| H0298-0221 | 0.5 | 0.5 | 0.125 | 0.125 |
| 334756 | 0.5 | 0.5 | 0.5 | 0.5 |
| 565708x | 0.5 | 0.5 | 0.25 | 0.25 |
| M9925 | 0.25 | 0.25 | 0.5 | 0.5 |
| 53 | 0.5 | 0.5 | 0.125 | 0.5 |
| 3306585 | 0.5 | 0.5 | 0.125 | 0.125 |
| 562964 | 0.5 | 0.5 | 0.25 | 0.25 |
| 565350 | 0.5 | 0.5 | 0.5 | 0.5 |
| 666432 | 0.25 | 0.25 | 0.5 | 0.5 |
| 16232 | 0.5 | 0.5 | 0.25 | 0.25 |
| 18863 | 0.5 | 0.5 | 0.5 | 0.5 |
| 16656 | 0.25 | 0.25 | 0.5 | 0.5 |
| 18825 | 0.5 | 0.5 | 0.125 | 0.125 |
| 18870 | 0.5 | 0.5 | 0.25 | 0.25 |
| 17997 | 0.5 | 0.5 | 0.25 | 0.25 |
